# Educational technology to support patient safety in the operating room: clinical simulation guides *

**DOI:** 10.1590/1518-8345.7085.4368

**Published:** 2024-11-22

**Authors:** Letícia Marie Sakai, Neide da Silva Knihs, Ana Graziela Alvarez, Patrícia Treviso, Aline Lima Pestana Magalhães, Débora Cristina Popov

**Affiliations:** ^1^ Universidade Federal de Santa Catarina, Enfermagem, Florianópolis, SC, Brazil.; ^2^ Universidade do Vale do Rio dos Sinos, Enfermagem, São Leopoldo, RS, Brazil.; ^3^ Universidade Santo Amaro, Enfermagem, São Paulo, SP, Brazil.

**Keywords:** Educational Technology, Patient Safety, Simulation Training, Organizational Culture, Surgical Centers, Perioperative Nursing

## Abstract

to develop and validate two clinical simulation guides to support and disseminate a culture of safety in the surgical environment.

methodological study supported by the International Nursing Association for Clinical Simulation and Learning theoretical framework. The snowball technique was used to validate the clinical guidelines with experts on the subject. Two scales validated for Brazil were used for the pilot test.

89 operating room professionals took part, including 41 doctors, 40 nursing technicians, four nurses, two radiology technicians, and two nursing trainees. Two guides were created, one on conflict management and teamwork in patient safety during the transfer of the patient from the operating room to the post-anesthetic recovery room, and the other dealing with the assistance of the healthcare team in the transfer of care in the post-anesthetic recovery room. The evaluation of Satisfaction with Simulated Clinical Experiences had an average score of 8.3 and Satisfaction and Self-confidence in learning 4.1.

the guides have been validated and are suitable for replication in any surgical environment. We believe that this technology could contribute to improving the safety culture in the operating room by providing an opportunity for reflection and critical thinking.

## Highlights

**(1)** Clinical guidelines developed based on demand from participants.

**(2)** Production of two clinical guidelines applied in the operating room and recovery room.

**(3)** Validation of the clinical guidelines by experienced specialists in the field.

**(4)** High satisfaction rates among pilot test participants.

**(5)** Recognition of simulation as a strategy that supports a culture of safety.

## Introduction

 Educational technologies can promote the exchange of information, improve communication, and knowledge, and foster interaction and innovation in healthcare ^(^ 1 ^-^ 3 ^)^ . In this scenario of intense development of educational strategies, realistic simulations can provide immersion in scenarios, allowing for the understanding of facts, and misunderstood and unnoticed details that are similar to real work situations ^(^ 4 ^-^ 5 ^)^ . 

 Realistic simulation, like other educational technologies, supports and corroborates the improvement of technical and non-technical skills, creating opportunities to improve operational and multidisciplinary work, to the benefit of safe care in teaching and professional practice ^(^ 6 ^-^ 7 ^)^ . 

 A literature review on the use of simulation demonstrates the effectiveness of the use of simulation in health care in teaching and learning, and in developing skills, favoring the learning of students and health professionals ^(^ 8 ^)^ . 

 Simulation has made a major contribution during the novel coronavirus pandemic and is widely used in healthcare environments as an educational technology for training professionals. The use of this methodology has contributed to the development of essential skills for nurses, including critical thinking and technical skills ^(^ 9 ^-^ 10 ^)^ . 

 During the pandemic, various studies have used realistic simulation as an educational technology to improve surgical techniques ^(^ 11 ^-^ 13 ^)^ . Other studies have also shown the use of simulations focused on the preoperative period, i.e. drain care, patient identification, safe surgery, and simulation activities related to safe communication and health professional exposure to aerosol in the operating room ^(^ 11 ^-^ 13 ^)^ . 

 Based on the evidence found in the literature on the effectiveness of this type of educational technology, the study proposes the development and use of simulations to disseminate a culture of safety in the surgical environment. The justification for its use in the context of safety is based on its potential to promote the improvement of skills and stimulate the safety of healthcare staff, based on simulated environments. In addition, the benefits of using simulation in the surgical environment have been expressed in the improvement of clinical reasoning, communication, progress in initiative and decision-making related to work practices, self-confidence, and improvement of surgical techniques. Since this technology encourages participants to reflect on institutional protocols, it contributes to increased adherence to professional practice, as well as giving operating room managers the opportunity to test a multitude of possible changes in operating room management, without interrupting the ongoing workflow when using simulation ^(^ 14 ^-^ 16 ^)^ . 

 When considering the effectiveness of this educational technology in supporting training and disseminating best practices in the surgical environment, this tool has emerged as a strategy to bring about changes in safety in the surgical environment and, consequently, improvements in the safety culture. Some authors point out that simulation promotes an effective interpersonal relationship, which leads to an improvement in patient safety ^(^ 17 ^-^ 19 ^)^ . 

In view of the above, this study poses the following research question: what information is needed to compose clinical guides to promote the dissemination of safety culture in the surgical environment? The aim of this study was to develop and validate two clinical simulation guides to support and disseminate the safety culture in the surgical environment.

Based on realistic simulations, it is believed that professionals working in the surgical environment will have the opportunity to participate in realistic scenarios, where it is possible to evaluate their organization, performance, outcomes, and actions in the face of the reality of the safety culture.

## Method

### Type of study

 This is a methodological study, based on the Standards of Best Practice translated into Portuguese ^(^ 20 ^)^ , published by the International Nursing Association for Clinical Simulation and Learning (INACSL). 

### Study site

The study was carried out in a philanthropic hospital in the Itajaí Valley, in Santa Catarina, Brazil, which performs an average of 750 surgeries a month. The hospital has 115 inpatient beds and Intensive Care Units (ICU) (Adult, Pediatric, and Neonatal). The Surgical Center (SC) consists of eight operating rooms, six of which are equipped for video surgery. Different specialties are treated at the hospital, such as Orthopedics, Gynecology and Obstetrics, Neurology, Cardiology, Otorhinolaryngology, Urology, and Plastic Surgery, among others. The surgical team is multi-professional and is made up of anesthesiologists, surgeons from different specialties, nursing staff, and radiologists.

### Period

The study took place between April 2021 and February 2023.

### Study participants

Participants included professionals from the multi-professional team working in the SC of the participating institution, and other professionals with expertise in SC, clinical simulation, and patient safety, who validated the content of the clinical guidelines.

In total, the operating room has different professionals registered to work in the sector (50 surgeons, 15 anesthesiologists, 50 nursing technicians, four nurses, two radiology technicians, and two nursing trainees).

### Development of the methodological steps

**Figure 1:**
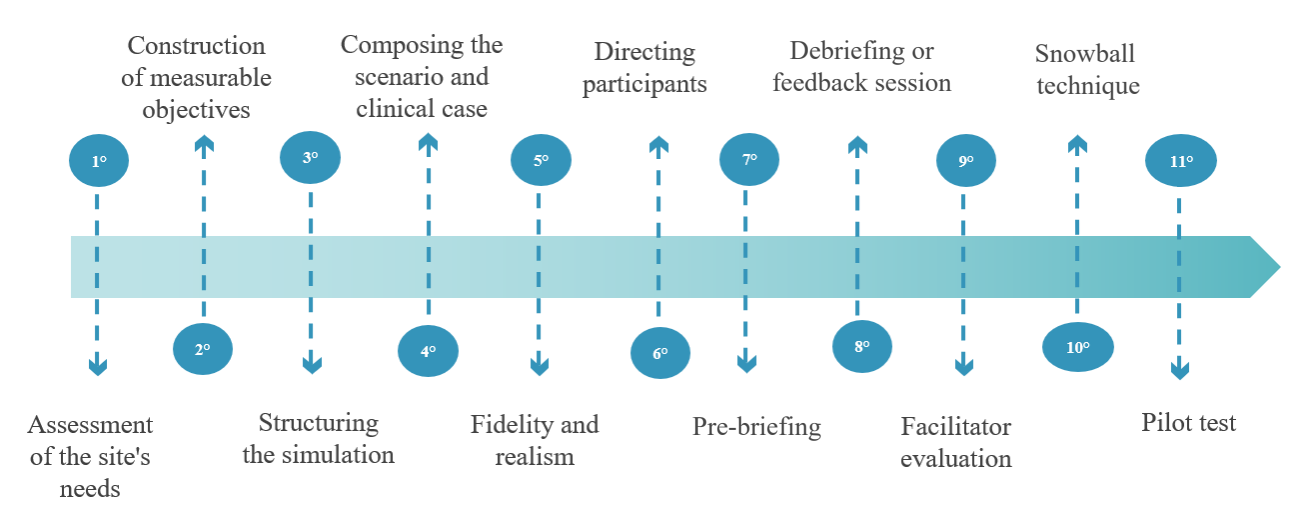
- Stages in the construction of clinical guidelines for simulation. Florianópolis, SC, Brazil, 2023

 The stages of the study follow the INACSL guidelines ^(^ 20 ^)^ , which include 11 criteria to be followed in the construction of clinical simulation guidelines ( Figure 1 ). 

*1st Criterion* - Assessment of the SC team’s needs - to build this criterion, a scoping review ^(^ 21 ^)^ was carried out, based on the method proposed by the Joanna Briggs Institute Reviewers ^(^ 22 ^)^ , to map out which educational strategies are most effective for disseminating surgical safety. 

In addition, the Hospital Survey on Patient Safety Culture (HSOPSC) questionnaire version 1.0 was used to measure the safety culture among the participating professionals.

 The HSOPSC ^(^ 23 ^)^ questionnaire, translated and adapted for Brazil, consists of 42 questions, divided into 12 dimensions: teamwork within the units; expectations and actions to promote patient safety by the supervisor/manager; organizational learning, continuous improvement; feedback and communication about errors; openness to communication; staffing; non-punitive responses to errors; support from hospital management for patient safety; teamwork between hospital units; internal transfers and duty shifts; general perception of patient safety and frequency of reported events. 

 The Agency for Healthcare Research and Quality (AHRQ) recommends that the questionnaire be evaluated using a Likert scale, with five points of agreement or frequency. It is recommended that the estimated average percentage of positive responses obtained in each dimension be used as a measure of the status of the safety culture. As an evaluation parameter, any dimension for which the percentage of positive responses is greater than or equal to 75% should be considered a strong or developed dimension of safety culture in the population studied. While dimensions with a percentage of positive responses less than or equal to 50% should be considered as potential for improvement and should be prioritized ^(^ 23 ^)^ . 

 However, it is important to note that the first studies that assessed safety culture in hospitals using the HSOPSC were not primarily focused on assessing safety culture, but on adapting the instrument cross-culturally for use in other countries. Many of these studies that assessed the dimensions of safety culture estimated average scores ranging from zero to five in each dimension, where an average score closer to 5.0 denotes a dimension in which the safety culture is strong among hospital staff ^(^ 23 ^)^ . 

The sample was calculated considering a significance level of 95%. The inclusion criteria were permanent members of the SC multi-professional team and, due to the high turnover of professionals, a minimum period of 30 days working in the sector was stipulated. The exclusion criteria were: professionals who did not belong to the SC, but who were working in the sector to cover vacations and/or leaves of absence, and professionals working in the sector who were on sick leave, leave of absence, or vacation during the data collection period.

Contact with the professionals took place at the workplace of one of the study’s researchers, after authorization from the hospital’s management and the SC. Data was collected using printed questionnaires from December 2022 to February 2023. The data was analyzed using statistical tests (absolute frequency, relative frequency, mean, median, standard deviation, interquartile range, parametric tests, and amplitude with Shapiro-Wilk normality test).

In an attempt to gain a deeper insight into the safety culture of the team taking part in the study, a document was created with six qualitative topics for brainwriting. This document was applied to six professionals, one nurse, and five nursing technicians, in a meeting held by one of the researchers in the SC’s training room, lasting approximately 40 minutes.

In each of the topics, the participant described three suggestions, individually, based on their vision and experience. The data was analyzed by similarity. The ideas that came up the most were organized into tables and word clouds on the Word Cloud platform. The guests who agreed to take part signed the Free and Informed Consent Term (FICT), one copy of which was given to each participant.

*2nd Criterion* - Construction of measurable objectives for the two clinical guidelines. At this stage, the researchers carefully analyzed the results obtained in the HSOPSC questionnaire and brainwriting to identify the greatest weaknesses. This led them to define the highest priority items and themes for the simulation guidelines, as well as the objectives for addressing these weaknesses. 

*3rd Criterion* - For the modality/structure of the simulation, the structure of the SC of the hospital where the data collection was carried out was considered, assessing the availability of existing materials in the institution for this purpose. 

*4th, 5th, and 6th Criteria* - Composition of the scenario and the patient’s clinical case according to the theme; enhancement and fidelity to the scene; a detailed description of the roles of each participant in the scene, directing them towards the proposed objectives. These criteria were grouped together, as they involved writing the clinical guide. For this stage, the weaknesses identified were taken into account, based on the proposed themes and objectives. The scientific literature related to the topic was also used as a basis. The composition of the scenario with the participants, roles, and speeches was reflected in the achievement of the clinical guide’s objectives and the fidelity of the scene. This stage was written by the researchers with the support of the supervisor, using a specific clinical guide template ^(^ 4 ^)^ . 

Once the first version of the guidelines had been written, they were sent for validation by professionals with experience in the subject. The snowball technique was used, considering an intentional random sample of three initial participants, who could indicate other participants.

The inclusion criteria were: having proven practical experience in SC, patient safety, and the use of simulation for more than five years; having published work on the topics: of SC, patient safety, and use of simulation. To make up the sample, at least two of the criteria had to be present.

 The professionals were identified by consulting their *Curriculum Lattes* (from National Council for Scientific and Technological Development – CNPq), using the keywords: simulation; organizational culture; perioperative nursing; patient safety; and educational technology. Those who agreed to take part in this stage of the study were contacted by e-mail and sent a FICT to sign, a clinical guide for validation, and a table for scoring the following scores: 1- keep the item; 2- keep with modifications; 3- delete the item; 4- add the item. When scoring 2, 3, or 4, the evaluators had to write the proposed change/exclusion/addition in the box next to the item being evaluated. 

The deadline set was 30 days for these professionals to make this assessment. After the files were returned with the suggestions, they were adjusted in the clinical guidelines, generating the second version. At the same time, the score data was tabulated in an electronic spreadsheet (Microsoft Excel) for descriptive statistics (mean, maximum/minimum, and median).

 The *7th, 8th, 9th, 10th,* and *11th criteria* were carried out sequentially by applying the pre-test. The venue for these stages was the SC (room 6). With regard to the participants, the sample was intentionally random, as the intention was to identify professionals from all shifts and the multi-professional team. The inclusion and exclusion criteria were the same as those described in *Criterion 1* . It is worth noting that the medical team was not available to take part in this activity. The professionals who agreed to take part in the simulation were given the FICT in two copies, one of which was given to the participant and the other to the researchers. After signing the FICT, a personalized date and time were scheduled for SC professionals to take part, depending on their availability. 

*7th Criterion* - Pre-briefing was carried out with the participants when they were informed about the themes of the simulations. The pre-briefing took place 10 minutes before the activity. The participants were briefed on the objectives of the scene, the skills to be developed, the time allotted, the equipment to be used, and whether the scene would be filmed. They were then given the presented case, from which they had five minutes to discuss the roles and the scene before the simulated scenario began. Each of the simulations had an actor, who had been previously instructed by the researchers the day before the simulation. 

 At the end of the scene, a debriefing ( *8th Criterion* ) took place in the same room as the pilot test, where the researchers talked to the participants about the items assessed in the scene and their performance, with the support of the examiner’s checklist. The participants were seated so that they could be comfortable as the recording of the scenes was projected and discussed, lasting 30 minutes. Initial questions included: How did you feel in the scene? What do you think you did that was interesting in the scene that is worth repeating? What do you think was not good about the scene and needs to be adjusted to improve it in practice? 

 The simulation facilitators ( *9th Criterion* ) included the researcher with training in simulation and the supervisor with more than 10 years of experience in simulation. For the *10th Criterion* , all the supporting documents and materials were provided so that the participants could achieve the objectives described in the annexes to the clinical guidelines. 

 For the *11th Criterion* , regarding the evaluation of the simulation, after the end of the debriefing the participants were given two printed scales, validated for the Brazilian context: the Learning Satisfaction and Self-Confidence Scale ^(^ 24 ^)^ and the Satisfaction with Simulated Clinical Experiences Scale ^(^ 25 ^)^ , answered using a Likert-type scale (scores from 1 to 10), where 1 means the lowest level of satisfaction and 10 the highest level of satisfaction. The data collected was compiled in an electronic spreadsheet (Microsoft Excel) and analyzed using descriptive statistics (mean, median, standard deviation). 

### Ethical aspects

The study was approved by the Ethics and Research Committee of the Federal University of Santa Catarina under CAAE (Certificate of Submission for Ethical Appraisal) No. 57625422.7.0000.0121 and Opinion No. 5.425.353. The anonymity of the participants was maintained at all stages of the study, as well as the confidentiality of the information collected, with access limited to the team taking part in the research.

## Results

 For the presentation of the results, the steps shown in the method will be considered in accordance with the INACSL ^(^ 20 ^)^ theoretical framework, in the form of graphs, tables, and figures. 

**Figure 2:**
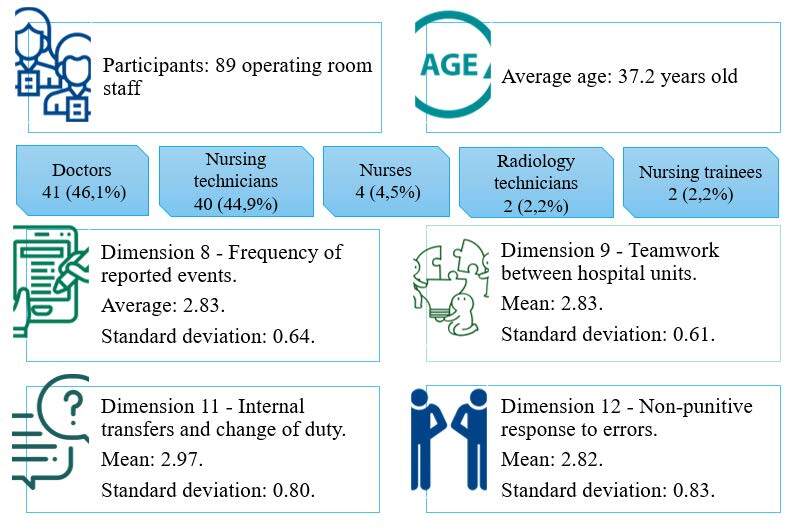
- Results of the Hospital Survey on Patient Safety Culture. Florianópolis, SC, Brazil, 2023

 The data obtained from the scoping review ( *1st Criterion* ) showed that simulation in high-fidelity controlled environments was the most prominent educational strategy capable of supporting the dissemination of a safety culture. Concerning the data obtained from the HSOPSC questionnaire, Figure 2 represents a summary of the results in terms of the characteristics of the participants, as well as the dimensions that obtained the lowest averages. 

Using the brainwriting technique, the main words present in the six themes indicated by the participants were: “hurry”, “lack of attention”, “training and orientation”, “pay more attention”, “more videos and training” and “more meetings with the team”.

**Figure 3:**
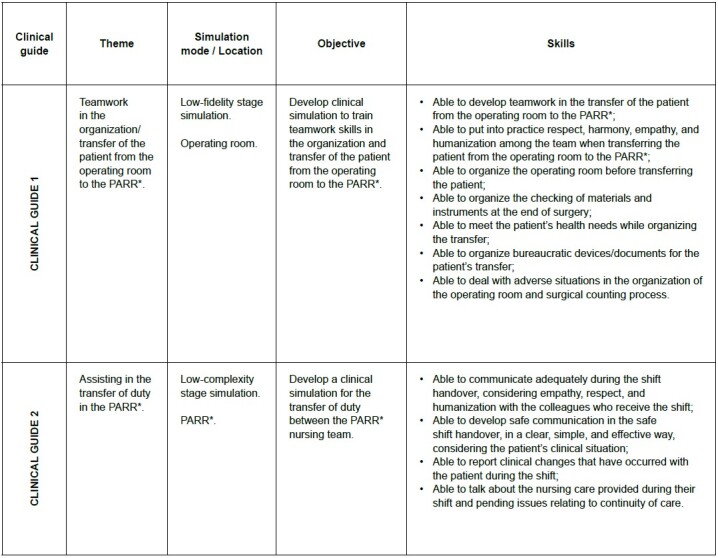
- Summary of the clinical guidelines developed. Florianópolis, SC, Brazil, 2023

 The results of *Criteria 2* and *3* were combined as they deal with the writing and construction of the objectives and structure of the clinical guideline. Figure 3 shows the main results relating to the objectives and structure of these clinical guidelines. The first column shows which guideline the text refers to (1 and 2). The second column refers to the topic, followed by the modality/type of simulation; the place where the simulation will be carried out, and the objectives and skills. It is worth noting that these two criteria were constructed taking into account the priorities identified in the *1st Criterion.*

*PARR = Post-Anesthetic Recovery Room

*Criteria 4, 5,* and *6* were grouped together because they refer to the step-by-step writing of the clinical guide. Due to the extent of the information in these criteria, they will be made available as complementary data. 

As for the data obtained during the validation of the clinical guidelines, nine professionals took part, eight of them nurses (88.9%) and one doctor (11.1%). Three of them had post-doctoral training (33.3%), three doctors (33.3%), two masters (22.2%), and one specialist (11.1%). As for their area of activity, six of them worked as teachers in surgery using simulations (66.6%) and three worked in SC care (33.3%). In addition, six of them had published on the subject. Their average length of experience was 26.5 years.

 The results of the validation of the clinical guidelines by these professionals, presented in Table 1 , show that most of them gave a score of 1 (keep the item) and 2 (keep the item with modifications). 

On the item Procedures (if any), one of the professionals (Professional 2) scored 4 (add item), suggesting adding new information. The average score for the items evaluated by all the experts was 1.35, with a median of 1 and a standard deviation of 0.25.

With regard to the evaluation of the complete simulated station, it can be seen that the majority of professionals scored 1 (keep the item) and 2 (keep with modifications). Professional 5 was the only one to score 3 (exclude the item) for the item: guidelines for the actress. Professional 9, on the other hand, scored 3 (exclude item) for the item: instruction for the scenario-description of the scenario and scored 4 (add item) for the participant’s resource. The mean score for the complete station was 1.34, median 1, and standard deviation 0.29.

*Criteria 7, 8, 9, 10,* and *11* , included in the pilot test stage, were carried out during the development of the simulated station with the multi-professional team in the surgical environment, with the participation of 19 professionals. The average age was 30.1 years, 94.7% female (n=18), the average time working in the hospital was 59 months, and in the SC 54 months. 

 Each of the simulations was developed taking into account the steps described in the guides. The clinics contain a lot of information, i.e. they describe the learning objectives, the materials to be used, the description of the scenario, the step-by-step speech of the actors, the flowchart of the scene, and the checklist to support the development of the debriefing. In this study, the authors chose to make the clinical guides available as supplementary material at the following link: https://doi.org/10.48331/scielodata.9X10QI . 

It is worth noting that each stage of the simulation was conducted as described in the method, using pre-briefing, briefing, debriefing, and the application of the Satisfaction with Simulated Experiences and Satisfaction and Self-Confidence in Learning scales.

Table 1 shows the results obtained by using the scale to assess Satisfaction with Simulated Experiences after the simulation had been developed by the participants. 

**Table 1:** - Results regarding Satisfaction with Simulated Experiences. Florianópolis, SC, Brazil, 2023

**Issues**	**Average**	SD*
1 ^st^ Overall satisfaction with the simulation.	7.2	2.5
2 ^nd^ Achievement of the simulation objective.	8.6	2.3
3 ^rd^ Dynamism of the simulation.	8.1	1.9
4 ^th^ Active participation in the scenario developed.	8.5	1.8
5 ^th^ Interaction with colleagues.	8.2	1.5
6 ^th^ Interaction with the nurse supervisor.	8.2	2.2
7 ^th^ Satisfaction with the degree of difficulty of the scenario.	7.9	2.1
8 ^th^ Satisfaction with the post-scenario discussion (debriefing).	9.5	0.7
9 ^th^ Linking the scenarios to theory.	8.7	1.9
10 ^th^ Realism of the scenario developed.	8.3	1.8
11 ^th^ Credibility during the scenario.	8.3	1.4
12 ^th^ Quality of the material used.	9.1	0.8
13 ^th^ Quality of the equipment.	9.2	0.8
**Overall average**	**8.3**	

* SD = Standard deviation

 The results of the evaluation according to the Learning Satisfaction and Self-Confidence Scale, using a Likert scale from 1 to 5, are shown in Table 2 . 

**Table 2:** - Results regarding Satisfaction and Self-confidence in learning. Florianópolis, SC, Brazil, 2023

**Issues**	**Average**	SD*
1 ^st^ The teaching methods used in this simulation were useful and effective.	4.5	0.5
2 ^nd^ The simulation provided a variety of teaching materials and activities to promote my learning of the medical-surgical curriculum.	4.3	0.5
3 ^rd^ I liked the way the nurse taught through the simulation.	3.8	1.4
4 ^th^ The teaching materials used in this simulation were motivating and helped me learn.	4.3	0.6
5 ^th^ The way the nurse taught through the simulation was appropriate for the way I learned.	4.0	1.2
6 ^th^ I am confident that I have mastered the content of the simulation activity that the nurse presented to me.	3.7	0.7
7 ^th^ I am confident that this simulation included the content necessary to master SC ^†^ care.	4.3	0.7
8 ^th^ I am confident that I am developing the necessary skills and knowledge from this simulation to perform the necessary procedures in a surgical environment.	4.5	0.5
9 ^th^ The nurse used useful resources to teach the simulation.	3.9	1.3
10 ^th^ It is my responsibility as a professional to learn what I need to know through the simulation activity.	4.5	0.6
11 ^th^ I know how to get help when I don’t understand the concepts covered in the simulation.	4.1	0.7
12 ^th^ I know how to use simulation activities to learn skills.	3.8	0.9
13 ^th^ It is the nurse’s responsibility to show me that I need to learn about the topic developed in the simulation.	3.3	0.9
**Média geral**	**4.1**	

* SD = Standard Deviation

^†^ SC = Surgical Center

The clinical guidelines presented and validated can be replicated in any surgical environment. Thus, the authors understand the importance of other professionals having access to these clinical guidelines.

## Discussion

 The study involved the participation of a multi-professional team at all stages of the development of the clinical guidelines. The average age of the participants was young adults, with doctors and nursing technicians participating in the same proportion. With regard to the participation of these professionals in assessing the safety culture, the results of other studies have pointed to this metric as being strategic and fundamental, since they are directly involved in the scenario of procedures in the surgical environment. It should also be noted that the participants were young, a period of life considered favorable for a high level of perception and understanding of patient safety culture ^(^ 26 ^-^ 27 ^)^ . 

The participation of professionals who practice in SC, both in the application stage of the HSOPSC questionnaire and in the brainwriting technique, helped to identify the needs and priorities in terms of safety culture in the context of the study since the data revealed the weakest dimensions through the items assessed and the grouped words that came up the most in the brainwriting. These results show how power, hierarchy, pressure, intolerance, and fear can prevent a culture of safety from being strengthened in healthcare environments.

 Lack of communication, inadequate attitudes, fear of punishment for errors, and haste in carrying out patient care activities were highlighted as the weaknesses and/or greatest demands on the team in terms of safety culture. Other studies corroborate these findings, revealing hierarchical rigidity, the high demand for assignments, and the pressure to carry out tasks, leading to adverse events and errors, as the factors that trigger a low safety culture ^(^ 28 ^-^ 29 ^)^ . 

However, it should be noted that these findings were fundamental to the construction of the clinical guidelines, as they enabled the researchers to get closer to the reality experienced in practice by this team. They were able to develop the guide’s writing and step-by-step approach, focusing on their real needs. This allowed both the objectives and the skills to trigger reflections and possible changes in this practice. Making it safer, more effective, and, above all, bringing the understanding that a culture of safety can be deepened by changing the attitudes and behaviors of those involved in the workplace. Thus, minimizing the fear and insecurity of punishment.

 From this perspective, we found that the construction of clinical guidelines is in line with studies that have already used this educational strategy. The authors emphasized the importance of systematic planning to structure the simulated practices, with the aim of supporting the conduct and evaluation of the planned activities ^(^ 30 ^)^ . 

 In this way, the planning and construction of clinical guidelines guide the simulation in its execution and are pointed out as fundamental strategies for establishing good practices. In this study, each of the stages proposed in the theoretical framework was followed, resulting in properly structured clinical guidelines at each writing stage. In view of the fact that these instruments will improve the skills of the multi-professional team in the surgical setting. This is a complex environment that requires frequent training, based on scenarios that are close to day-to-day practice. It is worth noting that the clinical guidelines are categorized as management guidelines, which help with the assembly and maintenance of simulators and scenarios and are accessible to laboratory logistics. In this sense, authors emphasize that clinical guides need to articulate knowledge and highlight the scenario precisely in order to provide learning opportunities ^(^ 20 ^,^ 31 ^-^ 33 ^)^ . 

 Some studies point out that clinical guides need to represent scenarios that reproduce real practice, i.e. as truthfully as possible, and thus enable individuals to experience learning moments that stimulate critical thinking, problem-solving, and clinical decision-making. It is also necessary to clearly define the teaching/learning objectives, thus enabling the process of developing clinical simulation scenarios to take place following the content previously worked on ^(^ 34 ^-^ 37 ^)^ . 

In this way, it is understood that the two clinical guidelines developed in this study met the recommendations of the theoretical framework adopted and the literature consulted, considering the detail of the descriptions, the proposed objectives, skills, clinical case, actors’ speech, distractors, as well as the checklist for conducting the debriefing.

 It should be noted that each stage of the clinical guidelines is related to the data collected by the team in the *1st Criterion* of the theoretical framework adopted. These statements reinforce the results obtained during validation using the snowball technique. Score 1 (keep the item) was predominant, even when faced with experienced professionals with significant involvement in the subject, both in the surgical environment and in simulation and safety culture. However, it is worth noting that the participation of professionals promoted the improvement and validation of the educational technology developed. In this way, the items were improved and all the information was validated, under the critical eye of professionals with expertise in the subject, thus allowing the content to be refined, so that it can be used by other multi-professional teams in surgical environments, making this surgical space safer and more harmonious, in the sense of providing a welcoming relationship between people, without mistrust, insecurity and fear of revealing errors or failures. 

 The participation of experts on the subject supports the statements made by other authors, reiterating the importance of these tools in bringing realism to scenarios, and encouraging participants to improve their skills so as not to fear mistakes ^(^ 20 ^,^ 31 ^-^ 33 ^)^ . Thus, it is possible to understand that the participation of these professionals in the process of validating the clinical guidelines could help to disseminate a culture of safety in the surgical environment. 

 As for the participants’ satisfaction with the pre-test and debriefing, it was presented as the activity with the greatest satisfaction among the participants. In this study, the debriefing proved to be very satisfactory due to the lightness with which the reflections between the team and the researchers were conducted. Various studies have shown that the debriefing stage is one of the most important stages of a simulation, where the competencies and objectives sought are connected and reflected upon in the actions carried out ^(^ 20 ^,^ 24 ^,^ 38 ^)^ . This stage provides an opportunity to create new theoretical and/or practical knowledge, as well as a moment of self-reflection and self-learning, based on numerous development methods related to the simulation’s objective ^(^ 20 ^,^ 24 ^,^ 33 ^,^ 38 ^)^ . 

 As for the assessment of self-confidence in learning from simulated scenarios, the teaching methods used in this simulation were useful and effective, with the highest score coming in line with the satisfaction of the debriefing. In this way, the participants recognized the simulation as a strategy capable of promoting improvements in skills related to safety culture in practice. Another study that used the self-confidence scale obtained an average rating of between 3.51 and 4.12 (out of a score of up to five points), which concluded that the approach is a good strategy for promoting confidence during participation in simulated scenarios ^(^ 39 ^)^ . 

 This was corroborated by a study ^(^ 40 ^)^ , which observed an increase in the average value of the self-confidence domains after simulated cardiopulmonary resuscitation practices with nursing students. In this sense, self-confidence is considered to be an attitude related to the experiences replicated by individuals, who are more likely to perform interventions appropriately ^(^ 25 ^,^ 41 ^)^ . 

From the perspective of the methodological rigor followed in conducting this study, it is understood that the contributions are aimed at offering readers the step-by-step construction, validation, and development of the pre-test in simulated scenarios, capable of supporting awareness and critical reflection on the culture of safety in the surgical environment. It also points out that this educational technology could support nurses in clinical research in the surgical environment, in order to enhance the dissemination of the safety culture, as well as strengthen continuous education in healthcare spaces. At the same time, it has the potential to create environments for discussion and clinical reasoning capable of supporting the advancement of advanced practices in Brazil.

In terms of limitations, we would highlight the low level of adherence by medical staff to all the stages recommended by the theoretical framework. In addition to the low number of studies with high impact factors to promote discussion of the results.

## Conclusion

This study made it possible to develop two realistic simulation clinical guidelines on surgical safety, in accordance with the theoretical framework adopted, with the themes of Conflict management and teamwork for patient safety in the transfer of patients from the operating room to the PARR; and Assistance from the healthcare team in the transfer of care in the PARR.

These guides were developed to promote respect, empathy, humanization, harmony, assertive and safe handovers, identification of risks related to patient safety, and conflict management. The stages in the construction of these guides were based on the demands made by the participating professionals during the methodological path developed, to bring the simulated scenario as close as possible to the reality experienced daily, and thus provide participants with the opportunity to reflect personally on the simulation.

The guides developed were validated by experienced specialists in patient safety, SC, and clinical simulation, improving the educational technology and enabling adaptations for future applications in different realities.

The results of this study reinforce these findings, showing satisfaction rates among participants, and allowing realistic simulation to be recognized as a strategy capable of supporting, disseminating, and strengthening the safety culture in the SC.
