# Supplementary material for: Educational technology to support patient safety in the operating room: clinical simulation guides
Source: Rev Lat Am Enfermagem. 2024 Nov 22;32:e4368. doi: 10.1590/1518-8345.7085.4368 (PMC11654029; doi:10.1590/1518-8345.7085.4368)
Supplement: Translation

# Translation: Tecnología educativa para contribuir a la seguridad del paciente en el área quirúrgica: guías clínicas de simulación *

**DOI:** 10.1590/1518-8345.7085.4367

**Published:** 2024-10-24

**Authors:** Letícia Marie Sakai, Neide da Silva Knihs, Ana Graziela Alvarez, Patrícia Treviso, Aline Lima Pestana Magalhães, Débora Cristina Popov

**Affiliations:** ^1^ Universidade Federal de Santa Catarina, Enfermagem, Florianópolis, SC, Brasil.; ^2^ Universidade do Vale do Rio dos Sinos, Enfermagem, São Leopoldo, RS, Brasil.; ^3^ Universidade Santo Amaro, Enfermagem, São Paulo, SP, Brasil.

**Keywords:** Tecnología Educacional, Seguridad del Paciente, Entrenamiento de Simulación, Cultura Organizacional, Centros Quirúrgicos, Enfermería Perioperatoria

## Abstract

desarrollar y validar dos guías de simulación clínica que contribuyan y difundan la cultura de seguridad en el ambiente quirúrgico.

estudio metodológico basado en el marco teórico de la *International Nursing Association for Clinical Simulation and Learning* . Se aplicó la técnica de bola de nieve para validar las guías clínicas con expertos en el tema. Para la prueba piloto se utilizaron dos escalas validadas para Brasil.

participaron 89 profesionales del área quirúrgica, entre ellos 41 médicos, 40 técnicos en enfermería, cuatro enfermeros, dos técnicos de radiología y dos residentes de enfermería. Se crearon dos guías, una sobre manejo de conflictos y trabajo en equipo en seguridad del paciente durante el traslado del paciente del quirófano a la sala de recuperación posanestésica, y otra que abordaba la asistencia del equipo de salud en el traslado de la atención en la sala de recuperación posanestésica. La evaluación de Satisfacción con las Experiencias Clínicas Simuladas obtuvo una media de 8,3 y la Satisfacción y Autoconfianza en el Aprendizaje una de 4,1.

las guías están validadas y se pueden replicar en cualquier ambiente quirúrgico. Se considera que esta tecnología podría contribuir a mejorar la cultura de seguridad en el área quirúrgica a través de la oportunidad de reflexión y el pensamiento crítico.

## Destacados

**(1)** Guías clínicas desarrolladas en base a la demanda de los participantes.

**(2)** Elaboración de dos guías clínicas aplicadas en el quirófano y la sala de recuperación.

**(3)** Validación de guías clínicas por expertos experimentados en el área.

**(4)** Se obtuvieron altos índices de satisfacción de los participantes de la prueba piloto.

**(5)** La simulación fue reconocida como estrategia que contribuye a la cultura de seguridad.

## Introducción

 Las tecnologías educativas pueden promover el intercambio de información, mejorar la comunicación, el conocimiento, el saber y también fomentar la interacción y la innovación en salud ^(^ 1 ^-^ 3 ^)^ . En este escenario de intenso desarrollo de estrategias educativas, las simulaciones realistas pueden brindar la oportunidad de participar en escenarios que se parecen a situaciones reales de trabajo y permiten comprender los hechos, los detalles que no se entienden y pasan desapercibidos ^(^ 4 ^-^ 5 ^)^ . 

 La simulación realista, así como otras tecnologías educativas, contribuyen y fortalecen la mejora de habilidades técnicas y no técnicas, y crean oportunidades para mejorar el trabajo operativo y multidisciplinario, en pro de una atención segura en la enseñanza y la práctica profesional ^(^ 6 ^-^ 7 ^)^ . 

 Un estudio de revisión de la literatura sobre el uso de la simulación demuestra la efectividad del uso de la simulación en la atención para la salud en la enseñanza y el aprendizaje, y en el desarrollo de habilidades, que favorecen el aprendizaje de estudiantes y profesionales de la salud ^(^ 8 ^)^ . 

 La simulación realizó una gran contribución durante la pandemia del nuevo coronavirus, y fue ampliamente utilizada en ambientes de salud, como tecnología educativa, para la formación de profesionales. El uso de esta metodología contribuyó al desarrollo habilidades esenciales para el enfermero, que incluyen el pensamiento crítico y las habilidades técnicas ^(^ 9 ^-^ 10 ^)^ . 

 Durante la pandemia, diferentes estudios utilizaron la simulación realista como tecnología educativa para mejorar las técnicas quirúrgicas ^(^ 11 ^-^ 13 ^)^ . Además, otros estudios han demostrado el uso de simulaciones centradas en el período preoperatorio, es decir, cuidados con drenajes, identificación de pacientes, cirugía segura y actividades de simulación relacionadas con la comunicación segura y la exposición de los profesionales de la salud a aerosoles en el quirófano ^(^ 11 ^-^ 13 ^)^ . 

 En función de la evidencia encontrada en la literatura sobre la efectividad de este tipo de tecnología educativa, el estudio propone el desarrollo y uso de simulaciones para difundir la cultura de seguridad en el ambiente quirúrgico. La razón para usarla en el contexto de la seguridad es que tiene potencial para promover la mejora de las competencias y fomentar la seguridad del equipo de salud, a partir de ambientes simulados. Además, los beneficios del uso de la simulación en el ambiente quirúrgico se manifiestan en la mejora del razonamiento clínico, la comunicación, el progreso en la iniciativa y la toma de decisiones relacionadas con las prácticas laborales, la autoconfianza y el perfeccionamiento de las técnicas quirúrgicas. Dado que esta tecnología estimula la reflexión de los participantes sobre los protocolos institucionales, contribuye a que haya una mayor adherencia en la práctica profesional, además les permite a los responsables del quirófano probar una infinidad de posibles cambios en el manejo del quirófano, sin interrumpir el flujo de trabajo en curso, gracias al uso de la simulación ^(^ 14 ^-^ 16 ^)^ . 

 Al considerar la efectividad de esta tecnología educativa para colaborar con la capacitación y a difundir las mejores prácticas en el ambiente quirúrgico, esta herramienta surgió como una estrategia para realizar cambios en la seguridad en el ambiente quirúrgico y, por ende, mejorar la cultura de seguridad. Algunos autores destacan que la simulación promueve una relación interpersonal efectiva, lo que provoca una mejora en la seguridad del paciente ^(^ 17 ^-^ 19 ^)^ . 

Por lo tanto, este estudio plantea la siguiente pregunta de investigación: ¿qué información se necesita para elaborar guías clínicas que promuevan la difusión de una cultura de seguridad en el ambiente quirúrgico? Por ende, el objetivo de este estudio fue desarrollar y validar dos guías de simulación clínica que contribuyan y difundan la cultura de seguridad en el ambiente quirúrgico.

A partir de simulaciones realistas, se cree que los profesionales que actúan en el ambiente quirúrgico tendrán la oportunidad de participar en escenarios realistas, en los que se pueda evaluar la organización, el desempeño, los resultados y las acciones ante la realidad de la cultura de seguridad.

## Método

### Tipo de estudio

 Se trata de un estudio metodológico, basado en los Estándares de Mejores Prácticas traducidos al portugués ^(^ 20 ^)^ , publicado por la *International Nursing* *Association for Clinical Simulation and Learning* (INACSL). 

### Lugar del estudio

El estudio se realizó en un hospital filantrópico del Vale do Itajaí, en Santa Catarina, que realiza un promedio de 750 cirugías por mes. El hospital cuenta con 115 camas de internación y Unidades de Cuidados Intensivos (UCI) (Adultos, Pediátricos y Neonatales). El Centro Quirúrgico (CQ) está compuesto por ocho quirófanos, seis de los cuales están equipados para video cirugía. En el hospital se atienden diferentes especialidades, tales como: Ortopedia, Ginecología y Obstetricia, Neurología, Cardiología, Otorrinolaringología, Urología, Cirugía Plástica, entre otras. El equipo quirúrgico es multidisciplinario y está integrado por anestesistas, cirujanos de diferentes especialidades, equipos de Enfermería y Radiología.

### Periodo

El estudio se llevó a cabo entre abril de 2021 y febrero de 2023.

### Participantes del estudio

Participaron profesionales del equipo multidisciplinario que trabajaban en el CQ de la institución participante, y otros profesionales con experiencia en CQ, simulación clínica y seguridad del paciente, que fueron quienes validaron el contenido de las guías clínicas.

En total, el área quirúrgica cuenta con diferentes profesionales registrados para trabajar en el sector (50 cirujanos, 15 anestesiólogos, 50 técnicos en enfermería, cuatro enfermeros, dos técnicos de radiología y dos residentes de enfermería).

### Desarrollo de pasos metodológicos

 Las etapas de realización del estudio siguen las determinaciones de la INACSL ^(^ 20 ^)^ , que contemplan 11 criterios a seguir en la elaboración de guías clínicas de simulación ( Figura 1 ). 

**Figura 1:** - Pasos para la elaboración de guías clínicas para simulación. Florianópolis, SC, Brasil, 2023

*1* º *Criterio -* Evaluación de las necesidades del equipo del CQ - para elaborar este criterio se realizó una revisión de alcance ^(^ 21 ^)^ , basada en el método propuesto por el *Joanna* *Briggs Institute Reviewers* ^(^ 22 ^)^ , con el objetivo de mapear cuáles son las estrategias educativas más eficaz para difundir la seguridad quirúrgica. 

 Además, se aplicó el cuestionario *Hospital Survey on Patient Safety Culture* (HSOPSC) versión 1.0 para poder medir la cultura de seguridad de los profesionales participantes. 

 El cuestionario HSOPSC ^(^ 23 ^)^ traducido y adaptado para Brasil consta de 42 preguntas, distribuidas en 12 dimensiones: trabajo en equipo dentro de las unidades; expectativas y acciones para promover la seguridad del paciente del supervisor/gerente; aprendizaje organizacional, mejora continua; *feedback* y comunicación de errores; apertura a la comunicaci *ón* ; dimensionamiento del personal; respuestas no punitivas a los errores; apoyo de la dirección hospitalaria a la seguridad del paciente; trabajo en equipo entre las unidades hospitalarias; traslados internos y cambios de turno; percepción general de la seguridad del paciente y frecuencia de eventos reportados. 

 La *Agency for Healthcare Research and Quality* (AHRQ) recomienda que el cuestionario se evalúe mediante la escala *Likert* , con cinco puntos de concordancia o frecuencia. Se recomienda utilizar el porcentaje promedio estimado de respuestas positivas obtenidas en cada dimensión como medida del estado de la cultura de seguridad. Como parámetro evaluativo, cualquier dimensión para la cual el porcentaje de respuestas positivas sea mayor o igual al 75% hay que considerarla como una dimensión fuerte o desarrollada de la cultura de seguridad en la población estudiada. Mientras que las dimensiones con un porcentaje de respuestas positivas menor o igual al 50% hay que considerarlas como potenciales de mejora y deben priorizarse ^(^ 23 ^)^ . 

 Sin embargo, es importante destacar que los primeros estudios que evaluaron la cultura de seguridad en hospitales utilizando el HSOPSC no se centraron principalmente en evaluar la cultura de seguridad, sino en adaptar el instrumento transculturalmente para ser usado en otros países. Muchos de estos estudios que evaluaron las dimensiones de la cultura de seguridad estimaron puntuaciones medias que oscilaban entre cero y cinco en cada dimensión, en la que una puntuación media más cercana a 5,0 denota una dimensión en la cual los empleados del hospital tienen una cultura de seguridad fuerte ^(^ 23 ^)^ . 

La muestra se calculó considerando un nivel de significación del 95%. Los criterios de inclusión fueron profesionales permanentes del equipo multidisciplinario del CQ y debido a la alta rotación de profesionales se estipuló un período mínimo de 30 días de trabajo en el sector. Criterios de exclusión: profesionales que no pertenecen al CQ, pero que se encontraban trabajando en el sector, para cubrir vacaciones y/o licencias, y profesionales que trabajan en el sector que se estaban de licencia médica, algún otro tipo de licencia o vacaciones durante el período de recolección de datos.

El contacto con los profesionales se realizó en el entorno laboral de una de los investigadoras del estudio, con previa autorización de la dirección del hospital y del CQ. La recolección de datos se realizó mediante cuestionarios impresos de diciembre de 2022 a febrero de 2023. Los datos fueron analizados mediante pruebas estadísticas (frecuencia absoluta, relativa, media, mediana, desviación estándar, rango intercuartil, pruebas paramétricas, rango con prueba de normalidad de Shapiro-Wilk).

 Buscando profundizar la percepción de la cultura de seguridad del equipo que participa del estudio, se creó un documento con seis tópicos cualitativos, para aplicar el *brainwriting.* Este documento fue aplicado a seis profesionales, una enfermera y cinco técnicos en enfermería, en un encuentro realizado por una de las investigadoras en la sala de capacitación del CQ, con una duración aproximada de 40 minutos. 

 En cada uno de los tópicos, el participante describió tres sugerencias, de manera individual, basadas en su visión y experiencia. El análisis de los datos se produjo por similitud. Las ideas que más surgieron se organizaron en tablas y nubes de palabras en la plataforma *Word Cloud* . Los invitados que aceptaron participar firmaron el Formulario de Consentimiento Libre e Informado (FCLI), del cual se entregó una copia a cada participante. 

*2º Criterio* - Elaboración de los objetivos medibles de las dos guías clínicas. En esta etapa, las investigadoras analizaron cuidadosamente los resultados obtenidos en el cuestionario HSOPSC y en el *brainwriting,* buscando identificar las mayores debilidades mencionadas. De esa forma, se definieron los ítems que tenían mayor prioridad y los temas para elaborar las guías de simulación, así como los objetivos para superar esas debilidades. 

*3º Criterio* - Para la modalidad/estructura de la simulación se consideró la estructura del CQ del hospital donde se desarrolló la recolección de datos, y se evaluó la disponibilidad de materiales existentes en la institución para tal fin. 

*4º, 5º y 6º Criterios* - Composición del escenario y del caso clínico del paciente según la temática; perfeccionamiento y fidelidad de la escena; descripción detallada de los roles de cada participante en la escena, orientándolos hacia los objetivos propuestos. Estos criterios se agruparon, dado que forman parte de la redacción de la guía clínica. Para esta etapa se consideraron las debilidades identificadas, en base a los temas propuestos y objetivos. Además, se utilizó como base la literatura científica relacionada con el tema. La composición del escenario con los participantes, los roles y los discursos se reflejó en el logro de los objetivos de la guía clínica y la fidelidad de la escena. Esta etapa fue redactada por las investigadoras con ayuda de la orientadora, utilizando un modelo de guía clínica específico ^(^ 4 ^)^ . 

Cuando se finalizó la redacción de la primera versión de las guías, se las enviaron a los profesionales con experiencia en el tema para que realizaran la validación. Se utilizó la técnica de bola de nieve, se consideró muestra aleatoria intencional con tres participantes iniciales, que podrían indicar otros participantes.

Se establecieron como criterios de inclusión: tener experiencia práctica comprobada en CQ, seguridad del paciente y uso de simulación mayor a cinco años; haber publicado trabajos sobre los temas: CQ, seguridad del paciente y uso de simulación. Para formar parte de la muestra deben contar con al menos dos de los criterios.

 Los profesionales fueron identificados mediante consulta del *Curriculum Lattes* (del Consejo Nacional de Desarrollo Científico y Tecnológico de Brasil – CNPq), con las palabras clave: simulación; cultura de la organización; enfermería perioperatoria; seguridad del paciente; tecnología educativa. Se contactó a través de correo electrónico a quienes aceptaron participar en esta etapa del estudio, y se les envió un Termo de Consentimiento Livre y Esclarecido (TCLE) para firmar, una guía clínica para validación y un cuadro para asignar los siguientes puntajes: 1- mantener el ítem; 2- mantener con modificaciones; 3- eliminar el ítem; 4- agregar el ítem. Cuando asignaban puntaje 2, 3 o 4, los evaluadores debían escribir en el cuadro, al lado del ítem evaluado, la modificación/exclusión/adición propuesta. 

 El plazo estipulado para que los profesionales realizaran dicha evaluación fue de 30 días. Una vez que devolvieron los archivos con las sugerencias, se realizaron los ajustes en las guías clínicas para crear la segunda versión. Paralelamente, los datos de los puntajes fueron tabulados en una hoja de cálculo electrónica ( *Microsoft Excel* ) para realizar estadísticas descriptivas (media, máximo/mínimo y mediana). 

 Los *Criterios 7°, 8°, 9°, 10°* y *11°* se realizaron de manera secuencial mediante la aplicación del pretest. El lugar para el desarrollo de estas etapas fue el CQ (sala 6). En cuanto a los participantes, la muestra fue aleatoria intencional, ya que la intensión era identificar a los profesionales de todos los turnos y al equipo multidisciplinario. Los criterios de inclusión y exclusión fueron los mismos que se aplicaron en el *Criterio 1* . Cabe señalar que el equipo médico no estuvo disponible para participar en esta actividad. Los profesionales que aceptaron participar en la simulación recibieron dos copias del TCLE, una para el participante y otra para los investigadores. Luego de la firma del TCLE se programó una fecha y hora para cada participante, para que los profesionales del CQ pudieran participar, según disponibilidad. 

*7º Criterio* - Se realizó un *prebriefing* con los participantes, en el que se les dio información sobre los temas de las simulaciones. El *prebriefing* se realizó durante 10 minutos antes de la actividad, y se les informaron: los objetivos de la escena, las habilidades que se iban a desarrollar, el tiempo esperado, el equipo que se iba a utilizar y la autorización para filmar la escena. Posteriormente se entregó el caso presentado y tuvieron cinco minutos para discutir los roles y la escena antes de que comenzara el escenario simulado. Cada una de las simulaciones contó con un actor, que el día anterior a la simulación recibió orientación por parte de las investigadoras. 

 Cuando termino la escena se llevó a cabo un *debriefing* ( *Criterio 8* ), en la misma sala donde se realizó la prueba piloto, en el cual las investigadoras hablaron con los participantes sobre los ítems evaluados en la escena y su desempeño, con ayuda de la *checklist* del examinador. Los participantes se sentaron, para que estuvieran cómodos, mientras se proyectaba y comentaba la grabación de las escenas, que duraron 30 minutos. Las preguntas iniciales incluyeron: ¿Cómo se sintieron en la escena? ¿Qué creen que hicieron que fue realmente interesante en la escena que vale la pena repetir? ¿Qué crees que no estuvo bien en la escena y debe ajustarse para mejorar en la práctica? 

 Las facilitadoras en la simulación ( *Criterio 9* ) incluyeron a la investigadora con formación en simulación y a la orientadora con más de 10 años de experiencia en simulación. Y, para el *Criterio 10* , se proporcionaron todos los documentos y material de apoyo para que los participantes pudieran alcanzar los objetivos descritos en los anexos de las guías clínicas. 

 Para realizar el *Criterio 11* , en lo que respecta a la evaluación de la simulación, después de finalizar el *debriefing* , se les entregaron a los participantes dos escalas impresas, validadas para el contexto brasileño: Escala de Satisfacción y Autoconfianza en el Aprendizaje ^(^ 24 ^)^ y Escala de Satisfacción con las Experiencias Clínicas Simuladas ^(^ 25 ^)^ , cuyas respuestas correspondían a una escala Likert (puntajes de 1 a 10), donde 1 significa el nivel más bajo de satisfacción y 10 el nivel más alto de satisfacción. Los datos recopilados se compilaron en una hoja de cálculo electrónica ( *Microsoft Excel* ) y se analizaron mediante estadística descriptiva (media, mediana, desviación estándar). 

### Aspectos éticos

El estudio fue aprobado por el Comité de Ética e Investigación de la Universidad Federal de Santa Catarina bajo CAAE n° 57625422.7.0000.0121 y Dictamen n° 5.425.353. El anonimato de los participantes se mantuvo durante todas las fases del estudio, así como la confidencialidad de la información recopilada, a la que solo tenía acceso el equipo que participaba en la investigación.

## Resultados

 Para presentar los resultados se considerarán los pasos descritos en el método según el marco teórico de la INACSL ^(^ 20 ^)^ , en formato de gráficos, tablas y figuras. 

 Los datos obtenidos en la revisión de alcance ( *Criterio 1* ) mostraron que la simulación en entornos controlados de alta fidelidad era la estrategia educativa más destacada que puede contribuir a difundir la cultura de seguridad. Con respecto a los datos obtenidos del cuestionario HSOPSC, la Figura 2 representa un resumen de los resultados sobre las características de los participantes, así como las dimensiones que obtuvieron las medias más bajas. 

**Figura 2 -:** Resultados de la aplicación del cuestionario *Hospital* *Survey on Patient Safety Culture.* Florianópolis, SC, Brasil, 2023

 Utilizando la técnica de *Brainwriting* , se obtuvieron las principales palabras presentes en los seis temas señalados por los participantes, a saber: “prisa”, “falta de atención”, “capacitación y orientación”, “prestar más atención”, “más vídeos y capacitación” y “más reuniones con el equipo”. 

 Los resultados de los *Criterios 2* y *3* se agruparon, dado que abordan la redacción y elaboración de los objetivos y la estructura de la guía clínica. La Figura 3 presenta los principales resultados relacionados con los objetivos y la estructuración de las guías clínicas. La primera columna muestra a qué guía se refiere el texto (1 y 2). La segunda columna se refiere a la temática, seguida de la modalidad/tipo de simulación; el lugar donde se desarrollará la simulación, los objetivos y habilidades. Cabe destacar que estos dos criterios se elaboraron teniendo en cuenta las prioridades identificadas en el *1º Criterio.*

**Figura 3 -:** Resumen de las guías clínicas desarrolladas. Florianópolis, SC, Brasil, 2023

*SRPA = Sala de Recuperación Posanestésica

 Se agruparon los *criterios 4, 5* y *6* considerando que se refieren a la redacción de la guía clínica paso a paso. Debido a la amplitud de la información contenida en estos criterios, se pondrá a disposición como datos complementarios. 

En lo que respecta a los datos obtenidos durante la validación de las guías clínicas, participaron nueve profesionales, ocho enfermeros (88,9%) y un médico (11,1%). Tres de ellos tenían formación posdoctoral (33,3%), tres tenían doctorado (33,3%), dos tenían maestría (22,2%) y uno especialidad (11,1%). En cuanto al área de actividad, seis trabajaban como docentes en el área quirúrgica con uso de simulaciones (66,6%) y tres trabajaban en asistencia en CQ (33,3%). Además, seis de ellos tenían publicaciones sobre el tema de investigación. Y el tiempo medio de experiencia era de 26,5 años de trabajo.

 Los resultados de la validación de las guías clínicas que realizaron dichos profesionales se presentan en la Tabla 1 , y demuestran que la mayoría le asignó el puntaje 1 (mantener el ítem) y el puntaje 2 (mantener el ítem con modificaciones). 

En el ítem Procedimientos (si los hubiera), uno de los profesionales (Profesional 2) le asignó el puntaje 4 (agregar ítem), y sugirió agregar nueva información. La media del puntaje de los ítems evaluados por todos los expertos fue 1,35, la mediana 1 y la desviación estándar 0,25.

En cuanto a la evaluación de la estación simulada completa, se observa que la mayoría de los profesionales le asignaron el puntaje 1 (mantener el ítem) y el puntaje 2 (mantener con modificaciones). El Profesional 5 fue el único que le asignó 3 (excluyendo el ítem) al ítem: pautas para la actriz. El Profesional 9, por su parte, le asignó 3 (excluyendo el ítem) al ítem: instrucción para el escenario-descripción del escenario y le asignó 4 (agregar ítem) al recurso del participante. La media de toda la estación fue de 1,34, la mediana 1 y la desviación estándar 0,29.

 En cuanto a los Criterios *7, 8, 9, 10* y *11* , incluidos en la etapa de la prueba piloto, se realizaron en el desarrollo de la estación simulada con el equipo multidisciplinario del ambiente quirúrgico, con la participación de 19 profesionales. La edad media era de 30,1 años, el 94,7% era de sexo femenino (n=18), el tiempo promedio de trabajo en la institución hospitalaria era de 59 meses y en el CQ de 54 meses. 

 Cada una de las simulaciones se realizó considerando los pasos descritos en las guías. Porque las clínicas tienen mucha información, es decir, describen los objetivos de aprendizaje, los materiales a utilizar, la descripción del escenario, el discurso de los actores paso a paso, el diagrama de flujo de la escena y la *checklist* que ayuda a realizar el *debriefing.* En este estudio, los autores eligieron que las guías clínicas estuvieran disponibles como material complementario en el *link* : https://doi.org/10.48331/scielodata.9X10QI . 

 Cabe destacar que cada etapa de la simulación se realizó según lo descrito en el método mediante *prebriefing* ; *briefing* , *debriefing* y aplicando las escalas de Satisfacción con las Experiencias Simuladas y de Satisfacción y Autoconfianza en el Aprendizaje. 

 La Tabla 1 presenta los resultados obtenidos mediante el uso de la escala para evaluar la Satisfacción con las Experiencias Simuladas después de que los participantes realizaran la simulación. 

**Tabla 1 -:** Resultados sobre la Satisfacción con las Experiencias Simuladas. Florianópolis, SC, Brasil, 2023

**Preguntas**	**Media**	DE*
1º Satisfacción general con la simulación.	7,2	2,5
2º Lograr el objetivo de la simulación.	8,6	2,3
3º Dinamismo de la simulación.	8,1	1,9
4º Participación activa en el escenario desarrollado.	8,5	1,8
5º Interacción con los compañeros.	8,2	1,5
6º Interacción con la enfermera orientadora.	8,2	2,2
7º Satisfacción con el grado de dificultad del escenario.	7,9	2,1
8º Satisfacción con la discusión posterior al escenario ( *debriefing* ).	9,5	0,7
9º Relación entre los escenarios y la teoría.	8,7	1,9
10º Realismo del escenario desarrollado.	8,3	1,8
11º Credibilidad durante el escenario.	8,3	1,4
12º Calidad del material utilizado.	9,1	0,8
13º Calidad del equipamiento.	9,2	0,8
**Media general**	**8,3**	

* DE = Desviación estándar

 Los resultados de la evaluación según la Escala de Satisfacción y Autoconfianza en el Aprendizaje, considerando la escala Likert del 1 al 5, se presentan en la Tabla 2 . 

**Tabla 2 -:** Resultados sobre la Satisfacción y Autoconfianza en el Aprendizaje. Florianópolis, SC, Brasil, 2023

**Preguntas**	**Media**	DE*
1º Los métodos de enseñanza utilizados en esta simulación fueron útiles y efectivos.	4,5	0,5
2º La simulación proporcionó una variedad de materiales didácticos y actividades para promover mi aprendizaje del plan de estudios médico quirúrgico.	4,3	0,5
3º Me gustó la forma en que la enfermera enseñaba a través de la simulación.	3,8	1,4
4º Los materiales didácticos utilizados en esta simulación fueron motivadores y me ayudaron a aprender.	4,3	0,6
5º La forma en que la enfermera enseñó mediante simulación era apropiada para mi forma de aprender.	4,0	1,2
6º Estoy seguro que domino el contenido de la actividad de simulación que me presentó la enfermera.	3,7	0,7
7º Estoy seguro de que esta simulación incluyó el contenido necesario para dominar la atención en el CQ **†** .	4,3	0,7
8º Estoy seguro de que estoy desarrollando habilidades y adquiriendo el conocimiento necesario a partir de esta simulación para realizar los procedimientos necesarios en un ambiente quirúrgico.	4,5	0,5
9º La enfermera utilizó recursos útiles para enseñar la simulación.	3,9	1,3
10º Es mi responsabilidad como profesional aprender lo que necesito saber a través de la actividad de simulación.	4,5	0,6
11º Sé cómo obtener ayuda cuando no entiendo los conceptos abordados en la simulación.	4,1	0,7
12º Sé cómo utilizar actividades de simulación para aprender habilidades.	3,8	0,9
13º Es responsabilidad de la enfermera demostrar que necesito aprender sobre el tema desarrollado en la simulación.	3,3	0,9
**Media general**	**4,1**	

* DE = Desviación estándar

^†^ CQ = Centro quirúrgico

Las guías clínicas presentadas y válidas se pueden replicar en cualquier ambiente quirúrgico. Por ello, los autores entienden la importancia de que otros profesionales tengan acceso a estas guías clínicas.

## Discusión

 El estudio contó con la participación del equipo multidisciplinario en todas las etapas del desarrollo de las guías clínicas. En relación a la edad promedio de los participantes, eran adultos jóvenes, hubo la misma proporción de médicos y técnicos en enfermería. En cuanto a la participación de estos profesionales en la evaluación de la cultura de seguridad, los resultados de otros estudios indican que esta métrica es estratégica y fundamental, ya que están directamente involucrados en el escenario de los procedimientos en el ambiente quirúrgico. Además, se destaca que los participantes son jóvenes, y ese es un período de la vida que se considera favorable para que alcancen una alta percepción y comprensión de la cultura de seguridad del paciente ^(^ 26 ^-^ 27 ^)^ . 

 La participación de profesionales que experimentan la práctica del CQ, tanto en la etapa de aplicación del cuestionario HSOPSC como en la técnica de *brainwriting* , contribuyó para identificar las necesidades y prioridades en materia de cultura de seguridad en el contexto de estudio mencionado, ya que los datos revelaron cuáles eran las dimensiones con más débil idades a través de los ítems evaluados y de las palabras agrupadas que más sugieren en el *brainwriting* . Estos resultados demuestran cómo el poder, la jerarquía, la presión, la intolerancia y el miedo pueden impedir el fortalecimiento de la cultura de seguridad en los ambientes de salud. 

 La falta de comunicación, actitudes inadecuadas, miedo al castigo por errores y prisa al realizar las actividades de atención al paciente, se destacaron como debilidades y/o mayor demanda del equipo en relación a la cultura de seguridad. Otros estudios corroboran estos hallazgos, al revelar que los factores desencadenantes de una baja cultura de seguridad son rigidez jerárquica, alta demanda de trabajo, presión para realizar las tareas, que generan eventos adversos y errores ^(^ 28 ^-^ 29 ^)^ . 

Sin embargo, hay que destacar que esos hallazgos fueron fundamentales para elaborar las guías clínicas porque les permitieron a las investigadoras aproximarse a la realidad que vive el equipo en la práctica. Y, de esa forma, llevar a cabo la redacción y la guía paso a paso, centrándose en sus necesidades reales. Esto permitió que tanto los objetivos como las habilidades desencadenaran reflexiones y posibles cambios en la práctica. Hacerla más segura, más eficaz y, sobre todo, comprender que la cultura de seguridad puede profundizarse cambiando las actitudes y el comportamiento de los que forman parte del ambiente laboral. Y, por ende, reducir la inseguridad y el miedo al castigo.

 Por lo tanto, se identificó que la elaboración de las guías clínicas coincide con los estudios que ya utilizaron esta estrategia educativa. En ellos, los autores destacaron la importancia de la planificación sistemática, para estructurar prácticas simuladas, con el objetivo de contribuir a la conducción y evaluación de las actividades planificadas ^(^ 30 ^)^ . 

 Por lo tanto, la planificación y elaboración de guías clínicas orientan la ejecución de la simulación, y se las considera estrategias fundamentales para establecer buenas prácticas. En este estudio se siguió cada uno de los pasos propuestos en el marco teórico, por lo que la redacción de cada etapa de las guías clínicas está estructura adecuadamente. Hay que tener en cuenta que dichos instrumentos mejorarán las competencias del equipo multidisciplinario en el ambiente quirúrgico. Un entorno complejo que requiere capacitación frecuente, basada en escenarios similares a los de la práctica cotidiana. Cabe destacar que las guías clínicas se consideran guías de manejo, que contribuyen al montaje y mantenimiento de simuladores y escenarios, y son accesibles para la logística del laboratorio. Por ello, los autores destacan que las guías clínicas deben articular el conocimiento y resaltar el escenario con precisión para brindar oportunidades de aprendizaje ^(^ 20 ^,^ 31 ^-^ 33 ^)^ . 

 Algunos estudios señalan que las guías clínicas deben representar escenarios que reproduzcan la práctica real, es decir, con la mayor precisión posible, y que les permitan a los individuos vivir momentos de aprendizaje que estimulen el razonamiento crítico, la resolución de problemas y la toma de decisiones clínicas. También es necesario definir claramente los objetivos de enseñanza/aprendizaje, permitiendo así que el proceso de desarrollo de escenarios de simulación clínica se produzca de acuerdo con los contenidos previamente trabajados ^(^ 34 ^-^ 37 ^)^ . 

 Por lo tanto, se considera que las dos guías clínicas desarrolladas en este estudio cumplieron con las recomendaciones del marco teórico adoptado y de la literatura consultada, debido al detalle de las descripciones, los objetivos propuestos, las habilidades, el caso clínico, el discurso de los actores, los distractores, así como la *checklist* para realizar el *debriefing* . 

 Hay que destacar que cada etapa de las guías clínicas está relacionada con los datos recopilados por el equipo en el *1º Criterio* del marco teórico adoptado. Las afirmaciones refuerzan los resultados obtenidos durante la validación con la técnica de bola de nieve; en la que predominó el puntaje 1 (mantener el ítem), incluso ante profesionales experimentados y con importante participación en el tema, tanto en el ambiente quirúrgico como en la simulación y cultura de seguridad. Sin embargo, cabe resaltar que la participación de profesionales permitió mejorar y validar la tecnología educativa desarrollada. De esta manera, se perfeccionaron los ítems y se validó toda la información, según la mirada crítica de profesionales con experiencia en el tema, lo que permitió así pulir el contenido, para que pueda ser utilizado por otros equipos multidisciplinarios en ambientes quirúrgicos y hacer más seguro y armonioso este espacio quirúrgico, en el sentido de permitir que haya una mejor relación entre las personas, sin que exista desconfianza, inseguridad y miedo a revelar errores o fallas. 

 La participación de expertos en el tema respalda las afirmaciones de otros autores, que insisten en lo importante que son estas herramientas para que los escenarios sean realistas e incentiven a los participantes a mejorar su competencia para que no les teman a los errores ^(^ 20 ^,^ 31 ^-^ 33 ^)^ . De esa forma, es posible comprender que la participación de estos profesionales en el proceso de validación de guías clínicas puede contribuir a la difusión de la cultura de seguridad en el ambiente quirúrgico. 

 En cuanto a la satisfacción de los participantes en el pretest y el *debriefing* , fue la actividad con mayor satisfacción para los participantes. En este estudio, el *debriefing* resultó bastante satisfactorio debido a la naturalidad con la que se llevaron a cabo las reflexiones entre el equipo y los investigadores. Diferentes estudios indican que la etapa de *debriefing* es una de las etapas más importantes de una simulación, dado que es donde ocurre la conexión y reflexión entre las competencias y los objetivos propuestos y las acciones realizadas ^(^ 20 ^,^ 24 ^,^ 38 ^)^ . Esta etapa brinda la oportunidad de crear nuevo conocimiento teórico y/o práctico, y también un momento de autorreflexión y autoaprendizaje, a partir de muchos métodos de desarrollo, relacionados con el objetivo de la simulación ^(^ 20 ^,^ 24 ^,^ 33 ^,^ 38 ^)^ . 

 Con respecto a la autoevaluación de la confianza en el aprendizaje a partir de escenarios simulados, los métodos de enseñanza utilizados en esta simulación fueron útiles y efectivos, dado que obtuvo la mejor puntuación al igual que la satisfacción con el *debriefing* . De esta manera, los participantes reconocieron la simulación como una estrategia que puede promover mejoras en las competencias relacionadas con la cultura de seguridad en la práctica. Otro estudio que utilizó la escala de autoconfianza obtuvo una calificación media en la escala entre 3,51 y 4,12 (sobre una puntuación de hasta cinco puntos), y concluyó que el enfoque es una buena estrategia para promover la confianza durante la participación en escenarios de simulación ^(^ 39 ^)^ . 

 De igual forma, un estudio ^(^ 40 ^)^ observó un aumento en el valor promedio de los dominios de autoconfianza a partir de prácticas simuladas en reanimación cardiopulmonar con estudiantes de enfermería. Por ende, la autoconfianza se considera una actitud relacionada con experiencias replicadas por los individuos, quienes, en su opinión, tienen más probabilidades de realizar intervenciones de manera adecuada ^(^ 25 ^,^ 41 ^)^ . 

Desde la perspectiva del rigor metodológico seguido para realizar este estudio, se considera que los aportes pretenden brindarles a los lectores el paso a paso de la elaboración, validación y desarrollo del pretest en escenarios simulados, que contribuyan a la concientización y la reflexión crítica para la cultura de seguridad en el ambiente quirúrgico. Además, se observa que la tecnología educativa presentada puede ayudar a los enfermeros en la investigación clínica en el ambiente quirúrgico, dado que puede aumentar la difusión de la cultura de seguridad y fortalecer la educación continua en los espacios de salud. A su vez, tiene el potencial de crear ambientes de discusión y razonamiento clínico que contribuyan el avance de las prácticas avanzadas en Brasil.

Se considera una limitación del estudio la baja adherencia del equipo médico en todas las etapas recomendadas por el marco teórico. Además del bajo número de estudios con altos factores de impacto para promover la discusión de los resultados.

## Conclusión

Este estudio permitió elaborar dos guías clínicas de simulación realista sobre seguridad quirúrgica, de acuerdo con el marco teórico adoptado, cuya temática fue el Manejo de conflictos y el trabajo en equipo para la seguridad del paciente en el traslado del paciente del quirófano a la SRPA; y Asistencia del equipo de salud en el traslado de la atención en la SRPA.

Estas guías fueron desarrolladas con el objetivo de promover respeto, empatía, humanización, armonía, cambio de turno asertivo y seguro, identificación de riesgos relacionados con la seguridad del paciente y, también, el manejo de conflictos. Las etapas de elaboración de estas guías se basaron en la demanda que presentaron los profesionales participantes, durante el recorrido metodológico desarrollado, con el objetivo de que el escenario simulado se parezca los más posible a la realidad que viven diariamente, y así, brindarles a los participantes la oportunidad de realizar reflexiones personales a partir de la simulación.

Las guías desarrolladas fueron validadas por expertos experimentados en los temas de seguridad del paciente, CQ, simulación clínica, y contribuyen a mejorar la tecnología educativa y permiten realizar adaptaciones para futuras aplicaciones en diferentes realidades.

Los resultados del presente estudio refuerzan estos hallazgos, dado que presentan índices de satisfacción de los participantes, que permiten que la simulación realista sea reconocida como una estrategia que puede apoyar, difundir y fortalecer la cultura de seguridad en el CQ.

# Translation: Tecnologia educacional para apoiar a segurança do paciente no bloco cirúrgico: guias clínicas de simulação *

**DOI:** 10.1590/1518-8345.7085.4369

**Published:** 2024-10-24

**Authors:** Letícia Marie Sakai, Neide da Silva Knihs, Ana Graziela Alvarez, Patrícia Treviso, Aline Lima Pestana Magalhães, Débora Cristina Popov

**Affiliations:** ^1^ Universidade Federal de Santa Catarina, Enfermagem, Florianópolis, SC, Brasil.; ^2^ Universidade do Vale do Rio dos Sinos, Enfermagem, São Leopoldo, RS, Brasil.; ^3^ Universidade Santo Amaro, Enfermagem, São Paulo, SP, Brasil.

**Keywords:** Tecnologia Educacional, Segurança do Paciente, Treinamento por Simulação, Cultura Organizacional, Centros Cirúrgicos, Enfermagem Perioperatória

## Abstract

desenvolver e validar duas guias clínicas de simulação para o apoio e a disseminação da cultura de segurança no ambiente cirúrgico.

estudo metodológico apoiado pelo referencial teórico de *International Nursing Association for Clinical Simulation and Learning.* A técnica bola de neve foi aplicada para a validação das guias clínicas junto às expertises na temática. Para o teste piloto foram utilizadas duas escalas validadas para o Brasil.

participaram 89 profissionais do bloco cirúrgico, sendo 41 médicos, 40 técnicos de Enfermagem, quatro enfermeiros, dois técnicos de radiologia e dois estagiários de Enfermagem. Foram construídas duas guias, uma sobre a gestão de conflitos e o trabalho em equipe na segurança do paciente durante a transferência do paciente da sala operatória para a sala de recuperação pós-anestésica, e outra que tratou da assistência da equipe de saúde na transferência do cuidado na sala de recuperação pós-anestésica. A avaliação da Satisfação com as Experiências Clínicas Simuladas obteve média 8,3 e a Satisfação e Autoconfiança na aprendizagem, 4,1.

as guias encontram-se validadas e aptas para serem replicadas em qualquer ambiente cirúrgico. Considera-se que essa tecnologia poderá contribuir para melhoria da cultura de segurança no bloco cirúrgico pela oportunidade de reflexão e pensamento crítico.

## Destaques

**(1)** Guias clínicas desenvolvidas com base na demanda dos participantes.

**(2)** Produção de duas guias clínicas aplicadas no centro cirúrgico e sala de recuperação.

**(3)** Validação das guias clínicas por especialistas experientes na área.

**(4)** Índices de satisfação elevados dos participantes do teste piloto.

**(5)** Reconhecimento da simulação como estratégia que apoia a cultura de segurança.

## Introdução

 Tecnologias educacionais podem promover a troca de informações, aprimorar a comunicação, os conhecimentos, os saberes, e ainda, favorecer a interação e a inovação em saúde ^(^ 1 ^-^ 3 ^)^ . Nesse cenário de intenso desenvolvimento de estratégias educacionais, as simulações realísticas são capazes de oportunizar a imersão em cenários, permitindo a compreensão de fatos, detalhes incompreendidos e despercebidos que se assemelham às situações reais de trabalho ^(^ 4 ^-^ 5 ^)^ . 

 A simulação realística, assim como as outras tecnologias educacionais apoiam e corroboram para o aprimoramento das competências técnicas e não técnicas, criando oportunidades de aprimorar o trabalho operacional e multidisciplinar, em benefício de um cuidado seguro no ensino e na prática profissional ^(^ 6 ^-^ 7 ^)^ . 

 Um estudo de revisão de literatura sobre o uso de simulação demonstra a eficácia do uso de simulação na assistência em saúde no ensino e na aprendizagem, e no desenvolvimento de habilidades, favorecendo o aprendizado de estudantes e profissionais de saúde ^(^ 8 ^)^ . 

 A simulação teve uma grande contribuição no decorrer da pandemia do novo coronavírus, sendo amplamente utilizada nos ambientes de saúde, enquanto tecnologia educacional, para o treinamento de profissionais. O uso dessa metodologia tem colaborado para o desenvolvimento de competências essenciais aos enfermeiros, incluindo o pensamento crítico e as habilidades técnicas ^(^ 9 ^-^ 10 ^)^ . 

 Diferentes estudos utilizaram, na pandemia, a simulação realística como tecnologia educacional para aprimorar as técnicas cirúrgicas ^(^ 11 ^-^ 13 ^)^ . Ainda, outros estudos demostraram o uso de simulações com foco no pré-operatório, ou seja, cuidados com drenos, identificação do paciente, cirurgia segura e atividades de simulação relacionadas à comunicação segura e à exposição do profissional de saúde ao aerossol na sala de cirurgia ^(^ 11 ^-^ 13 ^)^ . 

 A partir das evidências encontradas na literatura sobre a efetividade deste tipo de tecnologia educacional, o estudo propõe o desenvolvimento e o uso de simulações para disseminação da cultura de segurança no ambiente cirúrgico. A justificativa de sua utilização no contexto da segurança está pautada em sua potencialidade para promover o aprimoramento das competências e estimular a segurança da equipe de saúde, a partir de ambientes simulados. Além disso, os benefícios do uso da simulação no ambiente cirúrgico têm sido expressos na melhoria do raciocínio clínico, na comunicação, progresso na iniciativa e na tomada de decisões relacionadas às práticas do trabalho, autoconfiança e aprimoramento das técnicas cirúrgicas. Esta tecnologia estimula a reflexão dos participantes aos protocolos institucionais, contribuindo para o aumento da adesão na prática profissional, bem como oportuniza aos gerentes de salas de cirurgia testar uma infinidade de possíveis mudanças no gerenciamento da sala de operações, sem interromper o fluxo de trabalho em andamento quando utilizada a simulação ^(^ 14 ^-^ 16 ^)^ . 

 Considerando a efetividade desta tecnologia educacional para apoiar as capacitações e disseminar as melhores práticas no ambiente cirúrgico, tal ferramenta surgiu como uma estratégia para proporcionar as mudanças na segurança no ambiente cirúrgico e, consequentemente, as melhorias na cultura de segurança. Alguns autores destacam que a simulação promove uma efetiva relação interpessoal, o que desencadeia um aprimoramento da segurança do paciente ^(^ 17 ^-^ 19 ^)^ . 

Diante do exposto, este estudo traz como pergunta da pesquisa: quais informações são necessárias para compor as guias clínicas para promover a disseminação da cultura de segurança no ambiente cirúrgico? Assim, o objetivo deste estudo foi desenvolver e validar duas guias clínicas de simulação para o apoio e a disseminação da cultura de segurança no ambiente cirúrgico.

A partir das simulações realísticas, acredita-se que os profissionais que atuam no ambiente cirúrgico terão a oportunidade de participar de cenários realísticos, onde é possível avaliar sua organização, desempenho, desfechos e atuação frente à realidade da cultura de segurança.

## Método

### Tipo do estudo

 Estudo metodológico, sustentado nos Padrões de Melhores Práticas traduzidas para o português ^(^ 20 ^)^ , publicados pela *International Nursing* *Association for Clinical Simulation and Learning* (INACSL). 

### Local do estudo

O estudo foi realizado em um hospital filantrópico do Vale do Itajaí, em Santa Catarina, Brasil, que conta com a realização média de 750 cirurgias ao mês. O hospital possui 115 leitos de internação e Unidades de Terapia Intensiva (UTI) (Adulto, Pediátrica e Neonatal). O Centro Cirúrgico (CC) é composto por oito salas cirúrgicas, sendo que destas, seis estão equipadas para vídeo cirurgia. Diferentes especialidades são atendidas no hospital, como: Ortopedia, Ginecologia e Obstetrícia, Neurologia, Cardiologia, Otorrinolaringologia, Urologia, Cirurgia Plástica, entre outras. A equipe cirúrgica é multiprofissional, sendo composta por anestesistas, cirurgiões de diferentes especialidades, equipe de Enfermagem e de Radiologia.

### Período

O estudo ocorreu de abril de 2021 a fevereiro de 2023.

### Participantes do estudo

 Os participantes incluíram profissionais da equipe multiprofissional que atuavam no CC da instituição participante e outros profissionais com *expertise* em CC, simulação clínica e segurança do paciente, os quais validaram o conteúdo das guias clínicas. 

No total, o bloco cirúrgico possui diferentes profissionais cadastrados para atuar no setor (50 cirurgiões, 15 anestesiologistas, 50 técnicos de Enfermagem, quatro enfermeiros, dois técnicos de Radiologia e dois estagiários de Enfermagem).

### Desenvolvimento das etapas metodológicas

 As etapas da condução do estudo seguem as determinações da INACSL ^(^ 20 ^)^ , contemplando 11 critérios a serem seguidos na construção de guias clínicas de simulação ( Figura 1 ). 

**Figura 1 -:** Etapas de construção de guias clínicas para simulação. Florianópolis, SC, Brasil, 2023

*1º Critério* - Avaliação das necessidades da equipe do CC - para a construção deste critério foi realizada uma revisão de escopo ^(^ 21 ^)^ , sustentada no método proposto por *Joanna* *Briggs Institute Reviewers* ^(^ 22 ^)^ , com o objetivo de mapear quais estratégias educacionais são mais efetivas para a disseminação da segurança cirúrgica. 

 Adicionalmente, foi aplicado o questionário *Hospital Survey on Patient Safety Culture* (HSOPSC) versão 1.0, para possibilitar a mensuração da cultura de segurança junto aos profissionais participantes. 

 O questionário HSOPSC ^(^ 23 ^)^ traduzido e adaptado para o Brasil é composto por 42 questões, distribuídas em 12 dimensões: trabalho em equipe dentro das unidades; expectativas e ações de promoção da segurança do paciente do supervisor/gerente; aprendizado organizacional, melhoria contínua; *feedback* e comunicação a respeito de erros; abertura para as comunicações; dimensionamento de pessoal; respostas não punitivas aos erros; apoio da gestão hospitalar para a segurança do paciente; trabalho em equipe entre as unidades do hospital; transferências internas e passagens de plantão; percepção geral de segurança do paciente e frequência de eventos relatados. 

 A *Agency for Healthcare Research and Quality* (AHRQ) recomenda que a avaliação do questionário seja realizada por meio da escala de Likert, com a apresentação de cinco pontos de concordância ou frequência. Recomenda-se usar a porcentagem média estimada das respostas positivas obtidas em cada dimensão, como medida do *status* da cultura de segurança. Como parâmetro avaliativo, qualquer dimensão para a qual o percentual de respostas positivas seja maior ou igual a 75% deve ser considerada uma dimensão forte ou desenvolvida da cultura de segurança na população estudada. Enquanto que as dimensões com porcentagem de respostas positivas menor ou igual a 50% devem ser consideradas como potenciais para as melhorias, devendo ser priorizadas ^(^ 23 ^)^ . 

 No entanto, é importante destacar que os primeiros estudos que avaliaram a cultura de segurança em hospitais usando o HSOPSC não tinham como principal foco avaliar a cultura de segurança, mas adaptar o instrumento transculturalmente para o uso em outros países. Muitos desses estudos que avaliaram as dimensões da cultura de segurança estimaram pontuações médias variando de zero a cinco em cada dimensão, em que uma pontuação média mais próxima de 5,0 denota uma dimensão em que a cultura de segurança é forte entre os funcionários do hospital ^(^ 23 ^)^ . 

A amostra foi calculada considerando um índice de significância de 95%. Os critérios de inclusão foram os profissionais fixos da equipe multiprofissional do CC e devido à alta rotatividade dos profissionais, estipulou-se o período mínimo de atuação de 30 dias no setor. Os critérios de exclusão: profissionais que não pertencem ao CC, porém, que estivessem trabalhando no setor, para a cobertura de férias e/ou afastamentos, e os profissionais atuantes no setor que estivessem de licença médica, afastamento ou férias no período da coleta de dados.

O contato com os profissionais foi no ambiente de trabalho de uma das pesquisadoras do estudo, após a autorização da gerência do hospital e do CC. A coleta de dados ocorreu utilizando questionários impressos no período de dezembro de 2022 a fevereiro de 2023. Os dados foram analisados por meio de testes estatísticos (frequência absoluta, relativa, média, mediana, desvio-padrão, intervalo interquartílico, testes paramétricos, amplitude com teste de normalidade de Shapiro-Wilk).

 Ao buscar aprofundar a percepção sobre a cultura de segurança da equipe participante do estudo, foi criado um documento com seis tópicos qualitativos, para a aplicação do *brainwriting* . Este documento foi aplicado com seis profissionais, sendo um enfermeiro e cinco técnicos de Enfermagem, em um encontro realizado por uma das pesquisadoras na sala de capacitação do CC, com duração de aproximadamente 40 minutos. 

 Em cada um dos tópicos, o participante descreveu três sugestões, individualmente, com base em sua visão e vivência. A análise dos dados ocorreu por similaridade. As ideias que mais surgiram foram organizadas em quadros e nuvem de palavras na plataforma *Word Cloud* . Os convidados que aceitaram participar assinaram o Termo de Consentimento Livre e Esclarecido (TCLE), sendo uma via entregue a cada participante. 

*2º Critério* - Construção dos objetivos mensuráveis das duas guias clínicas. Nesta etapa, as pesquisadoras analisaram criteriosamente os resultados obtidos no questionário HSOPSC e *brainwriting* , buscando identificar as maiores fragilidades apontadas. Assim foram definidos os itens de maior prioridade e os temas para a composição das guias de simulação, bem como os objetivos para suprir estas fragilidades. 

*3º Critério* - Para a modalidade/estrutura da simulação considerou-se a estrutura do CC do hospital onde foi desenvolvida a coleta de dados, avaliando a disponibilidade dos materiais existentes na instituição para esta finalidade. 

*4º, 5º e 6º Critérios* - Composição do cenário e o caso clínico do paciente conforme a temática; aprimoramento e fidelidade à cena; descrição minuciosa dos papéis de cada participante da cena, direcionando-os para os objetivos propostos. Estes critérios foram agrupados, haja vista que envolvem a escrita da guia clínica. Para essa etapa consideraram-se as fragilidades identificadas, a partir dos temas propostos e os objetivos. Ainda, foi utilizado um embasamento da literatura científica relacionada à temática. A composição do cenário com os participantes, papéis e as falas foi refletida no alcance dos objetivos da guia clínica e na fidelidade da cena. Essa etapa foi escrita pelas pesquisadoras com apoio da orientadora, usando um determinado modelo de guia clínica ^(^ 4 ^)^ . 

 Ao término da escrita da primeira versão das guias, estas foram enviadas para a validação por profissionais com experiência na temática. Foi utilizada a técnica *snowball,* sendo considerada uma amostra aleatória intencional com três participantes iniciais, os quais poderiam indicar outros participantes. 

Foram estabelecidos os critérios de inclusão: ter vivência prática comprovada em CC, segurança do paciente e uso de simulação há mais de cinco anos; apresentar trabalhos publicados nas temáticas: CC, segurança do paciente e uso de simulação. Para compor a amostra, dois dos critérios, no mínimo, deveriam estar presentes.

 A identificação dos profissionais ocorreu por consulta ao *Curriculum* Lattes, do CNPq, com as palavras-chave: simulação; cultura organizacional; Enfermagem perioperatória; segurança do paciente; tecnologia educacional. Para aqueles que aceitaram participar desta etapa do estudo, foi realizado contato por *e-mail* , sendo enviados um Termo de Consentimento Livre e Esclarecido (TCLE) para assinar e uma guia clínica para a validação e um quadro para a pontuação dos seguintes escores: 1- manter o item; 2- manter com modificações; 3- excluir o item; 4- acrescentar o item. Quando assinalado os escores 2, 3 ou 4, os avaliadores deveriam escrever no quadro ao lado do item avaliado, a mudança/exclusão/acréscimo proposto. 

 O prazo determinado foi de 30 dias para que estes profissionais fizessem essa avaliação. Após o retorno dos arquivos com as sugestões, essas foram ajustadas nas guias clínicas, gerando a segunda versão. Concomitante a isso, os dados dos escores foram tabulados em uma planilha eletrônica ( *Microsoft Excel* ) para a realização de estatística descritiva (média, máximo/mínimo e mediana). 

 Os critérios 7º, 8º, 9º, 10º e 11º foram realizados sequencialmente por meio da aplicação do pré-teste. O local para o desenvolvimento destas etapas foi o CC (sala 6). Em relação aos participantes, a amostra foi aleatória intencional, haja vista que a intenção era identificar os profissionais de todos os turnos e a equipe multiprofissional. Os critérios de inclusão e exclusão foram os mesmos descritos no *Critério* *1º* . Vale ressaltar que não houve disponibilidade da equipe médica em participar desta atividade. Os profissionais que aceitaram participar da simulação receberam o TCLE em duas vias, sendo uma entregue ao participante e outra para as pesquisadoras. Após assinatura do TCLE foram agendados a data e o horário personalizados para que os profissionais do CC pudessem participar, conforme a disponibilidade. 

*7º Critério* - Pré- *briefing* foi realizado junto aos participantes, quando foram informados sobre os temas das simulações. O pré- *briefing* foi desenvolvido durante 10 minutos antes da atividade, sendo informado: os objetivos da cena, as habilidades a serem desenvolvidas, o tempo previsto, os equipamentos a serem utilizados e a concessão da filmagem da cena. Após, foi entregue o caso apresentado, e a partir desse momento os participantes tiveram cinco minutos para a discussão dos papéis e da cena antes do início do cenário simulado. Cada uma das simulações possuía um ator, que foi orientado previamente pelas pesquisadoras, no dia anterior à simulação. 

 Ao final da cena, ocorreu *debriefing* ( *Critério 8º* ) *,* realizado na mesma sala onde foi realizado o teste-piloto, no qual as pesquisadoras conversaram com os participantes a respeito dos itens avaliados na cena e seus desempenhos, com apoio do *checklist* do examinador. Os participantes foram acomodados sentados, para que pudessem estar confortáveis, à medida que a gravação das cenas fosse projetada e discutida, com duração de 30 minutos. As questões iniciais incluíram: Como vocês se sentiram na cena? O que vocês acham que fizeram de muito interessante na cena que vale a pena ser repetido? O que vocês acham que não ficou bom na cena e precisa ser ajustado para ser melhorado na prática? 

 As facilitadoras na simulação ( *Critério 9º* ) incluíram a pesquisadora com formação em simulação e a orientadora com experiência de mais de 10 anos em simulação. E, para o *Critério 10º* , houve o fornecimento de todos os documentos e materiais de apoio para que os participantes pudessem alcançar os objetivos descritos nos anexos das guias clínicas. 

 Para a realização do *Critério 11º* , quanto à avaliação da simulação, após o término do *debriefing* foram entregues aos participantes duas escalas impressas, validadas para o contexto brasileiro: Escala de Satisfação e Autoconfiança na Aprendizagem ^(^ 24 ^)^ e Escala de Satisfação com as Experiências Clínicas Simuladas ^(^ 25 ^)^ , respondidas por escala tipo Likert (escores de 1 a 10), onde 1 significa o menor nível de satisfação e 10 o maior nível de satisfação. Os dados coletados foram compilados em uma planilha eletrônica ( *Microsoft Excel* ) e analisados por estatística descritiva (média, mediana, desvio-padrão). 

### Aspectos éticos

A aprovação do estudo ocorreu pelo Comitê de Ética e Pesquisa da Universidade Federal de Santa Catarina sob CAAE n° 57625422.7.0000.0121 e Parecer n° 5.425.353. Manteve-se o anonimato dos participantes em todas as fases do estudo, bem como o sigilo das informações coletadas com o acesso limitado à equipe participante da pesquisa.

## Resultados

 Para a apresentação dos resultados serão consideradas as etapas exibidas no método conforme o referencial teórico da INACSL ^(^ 20 ^)^ , no formato de gráficos, quadros e figuras. 

 Os dados obtidos na revisão de escopo ( *Critério 1o* ) evidenciaram que a simulação em ambientes controlados de alta fidelidade foi a estratégia educacional mais destacada capaz de apoiar a disseminação da cultura de segurança. No que se refere aos dados obtidos do questionário HSOPSC, a Figura 2 representa uma síntese dos resultados quanto à característica dos participantes, bem como às dimensões que obtiveram as menores médias. 

**Figura 2 -:** Resultados da aplicação do questionário *Hospital* *Survey on Patient Safety Culture* . Florianópolis, SC, Brasil, 2023

 A partir da técnica do *brainwriting,* as principais palavras presentes nos seis temas indicados pelos participantes foram: “pressa”, “falta de atenção”, “treinamento e orientação”, “ter mais atenção”, “mais vídeos e treinamentos” e “mais reuniões com a equipe”. 

 Foram unidos os resultados dos *Critérios 2* e *3* , pois abordam a escrita e a construção dos objetivos e da estrutura da guia clínica. Na Figura 3 são apresentados os principais resultados relacionados aos objetivos e estruturação destas guias clínicas. Na primeira coluna mostra-se a qual guia se refere o texto (1 e 2). A segunda coluna refere-se ao tema, seguido da modalidade/tipo de simulação; o local onde será desenvolvida a simulação, os objetivos e habilidades. Vale salientar que esses dois critérios foram construídos levando em consideração as prioridades identificadas no *1º Critério.*

**Figura 3 -:** Síntese das guias clínicas desenvolvidas. Florianópolis, SC, Brasil, 2023

*SRPA = Sala de Recuperação Pós-Anestésica

 Realizou-se o agrupamento dos *Critérios 4, 5* e *6* , considerando que eles se referem à escrita do passo a passo da guia clínica. Devido à extensão das informações destes critérios, as mesmas serão disponibilizadas como dados complementares. 

Quanto aos dados obtidos durante a validação das guias clínicas, nove profissionais participaram, sendo oito enfermeiros (88,9%) e um médico (11,1%). Três deles possuíam formação de pós-doutorado (33,3%), três doutores (33,3%), dois mestres (22,2%) e um especialista (11,1%). Quanto à área de atuação, seis atuavam como professores na área cirúrgica com uso de simulações (66,6%) e três atuavam na assistência em CC (33,3%). Ainda, seis deles possuíam publicações na temática da pesquisa e seu tempo de experiência médio foi de 26,5 anos de atuação.

 Os resultados da validação das guias clínicas por estes profissionais, apresentados na Tabela 1 , demonstram que a maioria assinalou o escore 1 (manter o item) e o escore 2 (manter o item com modificações). 

 No item Procedimentos (se houver), um dos profissionais (Profissional 2) assinalou o escore 4 (acrescentar item), sugerindo acrescentar novas informações. A média dos escores entre os itens avaliados por todos os *experts* foi de 1,35, com mediana 1 e desvio-padrão 0,25. 

No que se refere à avaliação da estação simulada completa, nota-se que a maioria dos profissionais assinalaram os escores 1 (manter o item) e escore 2 (manter com modificações). O profissional 5 foi o único que pontuou o escore 3 (excluir o item) para o item: orientações para a atriz. Já o profissional 9, pontuou o escore 3 (excluir o item) para o item instrução para o cenário-descrição do cenário e assinalou escore 4 (acrescentar item) para recurso do participante. A média da estação completa foi de 1,34, mediana 1 e desvio-padrão 0,29.

 Quanto aos *Critérios* *7, 8, 9, 10* e *11* , incluídos na etapa do teste piloto, foram realizados no desenvolvimento da estação simulada junto à equipe multiprofissional do ambiente cirúrgico, com a participação de 19 profissionais. A média de idade foi de 30,1 anos, sexo feminino 94,7% (n=18), tempo médio de atuação na instituição hospitalar de 59 meses, e no CC 54 meses. 

 Cada uma das simulações foi desenvolvida considerando as etapas descritas nas guias. Em razão das clínicas possuírem muitas informações, haja vista que descrevem os objetivos de aprendizagem, os materiais a serem utilizados, a descrição do cenário, o passo a passo da fala dos atores, o fluxograma da cena e o *checklist* para apoiar o desenvolvimento do *debriefing.* Nesse estudo, os autores optaram por disponibilizar as guias clínicas como material suplementar no *link* : https://doi.org/10.48331/scielodata.9X10QI . 

 Vale destacar que cada etapa da simulação foi conduzida conforme descrição no método utilizando o pré- *briefing; briefing, debriefing* e aplicação das escalas de Satisfação com as Experiências Simuladas e Satisfação e Autoconfiança na aprendizagem. 

 Na Tabela 1 são apresentados os resultados obtidos por meio da utilização da escala para avaliar a Satisfação com as Experiências Simuladas após desenvolvimento da simulação pelos participantes. 

**Tabela 1 -:** Resultados quanto à Satisfação com as Experiências Simuladas. Florianópolis, SC, Brasil, 2023

**Questões**	**Média**	DP*
1º Satisfação global com a simulação.	7,2	2,5
2º Atinge o objetivo da simulação.	8,6	2,3
3º Dinamismo da simulação.	8,1	1,9
4º Participação ativa no cenário desenvolvido.	8,5	1,8
5º Interação com os colegas.	8,2	1,5
6º Interação com a enfermeira orientadora.	8,2	2,2
7º Satisfação com o grau de dificuldade do cenário.	7,9	2,1
8º Satisfação com a discussão pós-cenário ( *debriefing* ).	9,5	0,7
9º Ligação dos cenários à teoria.	8,7	1,9
10º Realismo do cenário desenvolvido.	8,3	1,8
11º Credibilidade durante o cenário.	8,3	1,4
12º Qualidade do material utilizado.	9,1	0,8
13º Qualidade dos equipamentos.	9,2	0,8
**Média geral**	**8,3**	

* DP = Desvio-padrão

 Os resultados da avaliação segundo a Escala de Satisfação e Autoconfiança na Aprendizagem, considerando a escala de Likert de 1 a 5, estão apresentados na Tabela 2 . 

**Tabela 2 -:** Resultados quanto a Satisfação e Autoconfiança na aprendizagem. Florianópolis, SC, Brasil, 2023

**Questões**	**Média**	DP*
1º Os métodos de ensino utilizados nesta simulação foram úteis e eficazes.	4,5	0,5
2º A simulação forneceu uma variedade de materiais didáticos e atividades para promover a minha aprendizagem do currículo médico cirúrgico.	4,3	0,5
3º Eu gostei do modo como a enfermeira ensinou por meio da simulação.	3,8	1,4
4º Os materiais didáticos utilizados nesta simulação foram motivadores e ajudaram-me a aprender.	4,3	0,6
5º A forma como a enfermeira ensinou por meio da simulação foi adequada para a forma como eu aprendo.	4,0	1,2
6º Estou confiante que domino o conteúdo da atividade de simulação que a enfermeira me apresentou.	3,7	0,7
7º Estou confiante que esta simulação incluiu o conteúdo necessário para o domínio do atendimento em CC ^†^ .	4,3	0,7
8º Estou confiante de que estou desenvolvendo habilidades e obtendo os conhecimentos necessários a partir desta simulação para executar os procedimentos necessários em um ambiente cirúrgico.	4,5	0,5
9º A enfermeira utilizou recursos úteis para ensinar a simulação.	3,9	1,3
10º É minha responsabilidade como profissional, aprender o que eu preciso saber por meio da atividade de simulação.	4,5	0,6
11º Eu sei como obter ajuda quando eu não entender os conceitos abordados na simulação.	4,1	0,7
12º Eu sei como usar atividades de simulação para aprender as habilidades.	3,8	0,9
13º É de responsabilidade da enfermeira mostrar que preciso aprender sobre a temática desenvolvida na simulação.	3,3	0,9
**Média geral**	**4,1**	

* DP = Desvio-padrão

^†^ CC = Centro cirúrgico

As guias clínicas apresentadas e validas podem ser replicadas em qualquer ambiente cirúrgico. Assim, os autores compreendem a importância de outros profissionais terem acesso a estas guias clínicas.

## Discussão

 O estudo contou com a participação da equipe multiprofissional em todas as etapas do desenvolvimento das guias clínicas. Em relação à idade média dos participantes, estes eram adultos jovens, com a participação de médicos e técnicos em Enfermagem na mesma proporção. No que se refere à participação destes profissionais na avaliação da cultura de segurança, resultados de outros estudos apontaram esta métrica como sendo estratégica e fundamental, visto que estão diretamente envolvidos no cenário dos procedimentos no ambiente cirúrgico. Ainda, destaca-se que os participantes são jovens, período de vida considerado favorável para a alta percepção e compreensão sobre a cultura de segurança do paciente ^(^ 26 ^-^ 27 ^)^ . 

 A participação dos profissionais que vivenciam a prática em CC, tanto na etapa da aplicação do questionário HSOPSC quanto na técnica do *brainwriting* , contribuiu para a identificação das necessidades e das prioridades quanto à cultura de segurança no referido contexto do estudo, visto que os dados revelaram as dimensões mais frágeis por meio dos itens avaliados, e das palavras agrupadas que mais sugiram no *brainwriting.* Tais resultados demonstram o quanto o poder, a hierarquização, a pressão, a intolerância e o medo podem impedir o fortalecimento da cultura de segurança nos ambientes de saúde. 

 A falta de comunicação, atitudes inadequadas, medo da punição aos erros e pressa em executar as atividades de cuidado ao paciente foram evidenciadas com as fragilidades e/ou maior demanda da equipe quanto à cultura de segurança. Outros estudos corroboram esses achados, ao revelar como fatores desencadeadores para uma baixa cultura de segurança a rigidez hierárquica, a elevada demanda de atribuições, a pressão em executar as tarefas implicando em eventos adversos e erros ^(^ 28 ^-^ 29 ^)^ . 

Todavia, destaca-se que tais achados foram fundamentais para a construção das guias clínicas em razão de possibilitarem às pesquisadoras aproximarem-se da realidade vivida na prática por essa equipe. E, assim, desenvolver a escrita e o passo a passo da guia focada nas reais carências deles. Isso permitiu que tanto os objetivos quanto às habilidades desencadeassem reflexões e possíveis mudanças desta prática. Tornando-a mais segura, efetiva e, principalmente, trazendo à compreensão de que a cultura de segurança pode ser aprofundada com as mudanças de atitudes e os comportamentos dos envolvidos no ambiente de trabalho. Assim, minimizando o medo e a insegurança da punição.

 Nessa perspectiva, identificou-se que a construção das guias clínicas vai ao encontro de estudos que já utilizaram esta estratégia educacional, em que os autores pontuam a importância do planejamento sistemático, para estruturar as práticas simuladas, com a finalidade de apoiar na condução e avaliação das atividades planejadas ^(^ 30 ^)^ . 

 Assim, o planejamento e a construção de guias clínicas norteiam a simulação em sua execução, sendo apontadas como estratégias fundamentais para o estabelecimento de boas práticas. Neste estudo, cada uma das etapas propostas no referencial teórico foi seguida, resultando, assim, em guias clínicas devidamente estruturadas em cada fase da escrita. Haja vista, que tais instrumentos proporcionarão o aprimoramento das competências da equipe multiprofissional do cenário cirúrgico. Um ambiente complexo que necessita de capacitações frequentes, embasadas em cenários que se aproximam da prática do dia a dia. Vale salientar que as guias clínicas são categorizadas como guias de manejo, as quais auxiliam a montagem e a manutenção dos simuladores e dos cenários, sendo acessíveis à logística do laboratório. Nesse sentido, autores destacam que as guias clínicas precisam articular o conhecimento e realçar o cenário com precisão no sentido de oportunizar aprendizagem ^(^ 20 ^,^ 31 ^-^ 33 ^)^ . 

 Alguns estudos apontam que as guias clínicas necessitam representar cenários que reproduzam a prática real, ou seja, com a maior veracidade possível, e, assim, possibilitar aos indivíduos a vivência de momentos de aprendizagem que estimulam o raciocínio crítico, solução de problemas e a tomada de decisão clínica. Sendo ainda necessária a definição clara dos objetivos de ensino/aprendizagem, possibilitando, assim, que o processo de desenvolvimento de cenários de simulações clínicas ocorra conforme os conteúdos trabalhados previamente ^(^ 34 ^-^ 37 ^)^ . 

 Desse modo, entende-se que as duas guias clínicas desenvolvidas neste estudo atenderam às recomendações do referencial teórico adotado e à literatura consultada, considerando o detalhamento das descrições, os objetivos propostos, habilidades, caso clínico, fala dos atores, distratores, bem como o *checklist* para conduzir o *debriefing* . 

 Destaca-se que cada etapa das guias clínicas se relaciona aos dados levantados pela equipe no *1º Critério* do referencial teórico adotado. As afirmações reforçam os resultados obtidos durante a validação com a técnica *snowball* , em que o escore 1 (manter o item) foi predominante, mesmo diante de profissionais experientes com envolvimento importante na temática, tanto em ambiente cirúrgico, quanto em simulação e cultura de segurança. No entanto, cabe destacar que a participação dos profissionais promoveu o aprimoramento e a validação da tecnologia educacional desenvolvida. Desse modo, os itens foram aperfeiçoados, bem como validadas todas as informações, sob o olhar crítico de profissionais com *expertise* na temática, permitindo, assim refinar o conteúdo, para que sejam utilizadas por outras equipes multiprofissionais nos ambientes cirúrgicos, tornando este espaço cirúrgico mais seguro e harmonioso, no sentido de proporcionar uma relação acolhedora entre as pessoas, sem que haja desconfiança, insegurança e medo de revelar erros ou falhas. 

 A participação de especialistas na temática apoia as afirmações de outros autores, reiterando a importância destas ferramentas de trazer realismo aos cenários, incentivando os participantes a aprimorarem a competência de maneira a não temer erros ^(^ 20 ^,^ 31 ^-^ 33 ^)^ . Assim, é possível compreender que a participação destes profissionais no processo de validação das guias clínicas possa colaborar para a disseminação da cultura de segurança no ambiente cirúrgico. 

 Quanto à satisfação dos participantes no pré-teste e *debriefing* , foi apresentada como a atividade com maior satisfação entre os participantes. Neste estudo, o *debriefing* se mostrou bastante satisfatório pela leveza com que foram conduzidas as reflexões entre a equipe e os pesquisadores. Diferentes estudos sinalizam que a etapa de *debriefing* é uma das etapas mais importantes de uma simulação, onde ocorre a conexão e a reflexão das competências e objetivos almejados com as ações executadas ^(^ 20 ^,^ 24 ^,^ 38 ^)^ . Nesta etapa é oportunizada a criação de novos conhecimentos, teóricos e/ou práticos, proporcionando também um momento de autorreflexão e autoaprendizagem, a partir de inúmeros métodos de desenvolvimento, relacionados ao objetivo da simulação ^(^ 20 ^,^ 24 ^,^ 33 ^,^ 38 ^)^ . 

 Quanto a avaliação da autoconfiança na aprendizagem a partir de cenários simulados, os métodos de ensino utilizados nesta simulação foram úteis e eficazes, sendo o melhor pontuado vindo ao encontro com a satisfação do *debriefing* . Desse modo, os participantes reconheceram a simulação como uma estratégia capaz de promover melhorias nas competências que se relacionam à cultura segurança na prática. Outro estudo que utilizou a escala de autoconfiança obteve classificação média da escala entre 3,51 a 4,12 (de uma pontuação de até cinco pontos), que concluiu que a abordagem é uma boa estratégia para a promoção de confiança durante a participação em cenários simulados ^(^ 39 ^)^ . 

 Corroborado por um estudo ^(^ 40 ^)^ , o qual observou um aumento do valor médio dos domínios de autoconfiança a partir de práticas simuladas em ressuscitação cardiopulmonar com acadêmicos de Enfermagem. Assim, nesse sentido, a autoconfiança é considerada uma atitude relacionada às experiências replicadas pelos indivíduos, que no que lhe concerne, apresentam maior probabilidade de executar as intervenções de modo adequado ^(^ 25 ^,^ 41 ^)^ . 

Na perspectiva do rigor metodológico seguido para condução deste estudo, compreende-se que as contribuições estão direcionadas a oferecer aos leitores o passo a passo da construção, validação e desenvolvimento do pré-teste em cenários simulados, capazes de apoiar a sensibilização e a reflexão crítica para a cultura de segurança em ambiente cirúrgico. Ainda, pontua-se que tal tecnologia educacional apresentada poderá apoiar enfermeiros em pesquisas clínicas no ambiente cirúrgico no sentido de potencializar a disseminação da cultura de segurança, bem como fortalecer a educação continuada nos espaços de saúde. Ao mesmo tempo que tem o potencial de criar ambientes de discussão e raciocínio clínico capazes de subsidiar o avanço das práticas avançadas no Brasil.

Quanto as limitações destacam-se a baixa adesão da equipe médica em todas as etapas preconizadas pelo referencial teórico. O baixo número de estudos com fatores de impacto elevado para promover a discussão dos resultados.

## Conclusão

Este estudo possibilitou desenvolver duas guias clínicas de simulação realística sobre a segurança cirúrgica, de acordo com o referencial teórico adotado, tendo como temática a Gestão de conflitos e o trabalho em equipe para a segurança do paciente na transferência do paciente da sala operatória para a SRPA; e a Assistência da equipe de saúde na transferência do cuidado na SRPA.

Tais guias foram desenvolvidas tendo por objetivo a promoção do respeito, empatia, humanização, harmonia, passagem de plantão assertiva e segura, identificação dos riscos relacionados à segurança do paciente e, ainda, a gerência de conflitos. As etapas da construção destas guias foram embasadas na demanda apresentada dos profissionais participantes, durante o percurso metodológico desenvolvido, de modo a aproximar ao máximo o cenário simulado da realidade vivenciada no cotidiano, e assim, oportunizar as reflexões pessoais dos participantes a partir da simulação.

As guias desenvolvidas foram validadas por especialistas experientes nas temáticas de segurança do paciente, CC, simulação clínica, proporcionando um aprimoramento da tecnologia educacional e possibilitando as adaptações para as futuras aplicações em diferentes realidades.

Os resultados deste estudo reforçam estes achados, apresentando índices de satisfação dos participantes, permitindo que a simulação realística seja reconhecida como uma estratégia capaz de apoiar, disseminar e fortalecer a cultura de segurança no CC.
